# Remote assessment of physical fitness via videoconferencing: a systematic review

**DOI:** 10.1186/s13102-024-01050-w

**Published:** 2025-01-23

**Authors:** Thorsten Klein, Annette Worth, Claudia Niessner, Anke Hanssen-Doose

**Affiliations:** 1 Institute of Movement and Sport, Karlsruhe University of Education, Bismarckstraße 10, 76133 Karlsruhe, Germany; 2 Institute of Sports and Sports Science, Karlsruhe Institute of Technology, Kaiserstraße 12, 76131 Karlsruhe, Germany

## Abstract

Physical fitness is a critical marker of overall health across all age groups, influencing longevity and quality of life. This systematic review assessed the use of remote physical fitness assessments, a crucial adaptation during the COVID-19 pandemic that broadened access to health monitoring outside traditional settings. The review included 35 studies, covering various age groups and health conditions, and evaluated 48 physical fitness tests across eight physical fitness components. Balance, muscular strength, and endurance were the most frequently assessed, with tests like the 30-second sit-to-stand (30s-STS) showing strong validity, reliability, and feasibility for remote use. However, the study population was mainly adults and older adults, with nearly no focus on children, revealing a significant gap in research for younger populations. Additionally, the review identified gaps in assessing components such as body composition, reaction time, and agility, which are crucial for a comprehensive assessment of physical fitness. These gaps underscore the need for further research and development of reliable and valid remote assessment tools. The findings of this review emphasize the importance of standardizing remote physical fitness assessments to ensure their validity, reliability, and feasibility making them effective tools for health monitoring across diverse populations and settings.

**Supplementary Information:**

The online version contains supplementary material available at 10.1186/s13102-024-01050-w.

## Introduction

Physical fitness is a crucial health marker for both current and future health status across all age groups, including children, adolescents, and adults [1–4]. Furthermore, being physical fit has been shown to positively influence longevity [5] and health-related quality of life [6]. Physical fitness can be divided into 11 components which fall into two groups [7]. The *health-related* components of physical fitness (a) cardiorespiratory endurance, (b) muscular endurance, (c) muscular strength, (d) body composition, (e) flexibility, and the *skill-related* components of (f) agility, (g) balance, (h) coordination, (i) speed, (j) power, and (k) reaction time [7]. The regular assessment of physical fitness provides essential insights into an individual’s overall health and potential risks for various conditions. In this context physical fitness tests are widely used to assess physical fitness, with different tests tailored to measure specific components of physical fitness. For instance, tests like the standing long jump or vertical jump are commonly used to assess the muscular strength of the lower body [8]. Conventionally, these assessments are conducted face-to-face in standardized settings, allowing a comprehensive test profile and the testing of a representative sample size [9]. However, the outbreak of the COVID-19 pandemic significantly disrupted this traditional approach, making it challenging to conduct face-to-face assessments in both scientific studies and healthcare settings. Consequently, remote delivery of physical fitness testing, defined as any non-face-to-face method including telephone, video, or postal delivery, has gained popularity.

The shift to remote delivery of physical fitness assessments presents both unique challenges and promising opportunities. Remote delivery can overcome logistical barriers such as transportation issues and social isolation. This broader accessibility can enhance the inclusion of underserved groups in clinical research [10] and ensure the continuity of studies during pandemic conditions. However, the digital divide [11] may exclude individuals lacking digital infrastructure or training, potentially leading to biases in study samples. Additionally, poor internet quality can lead to testing errors, affecting the accuracy and reliability of remote assessments. Therefore, it is important to rigorously evaluate the validity, reliability, and feasibility of remote testing methods.

To date, there has been limited systematic review of remote physical performance assessments. A systematic review by Heslop et al. (2023) [12] focused exclusively on older adults and investigated the agreement between face-to-face and remote assessments, as well as the feasibility of conducting remote assessments. In their review Heslop et al. (2023) [12] included nine different physical fitness measures and did not encompass the broader population or a wider range of physical fitness components [7]. Given the increasing reliance on remote methodologies, it is essential to expand the scope of research to include children, adolescents, and adults. Therefore, the aims of this systematic review are (1) to assess the evidence on how physical fitness is measured remotely using physical fitness tests and (2) to evaluate the validity, reliability, and feasibility of remote measuring methods for physical fitness across all age groups, starting from one year old. This review will provide new insights into the potential of remote physical fitness assessments to serve as reliable and valid alternatives to conventional face-to-face methods, thereby ensuring continuity and inclusivity in health research and practice.

## Methods

We conducted a systematic review following the criteria of the “Preferred Reporting Items for Systematic Reviews and Meta-Analyses (PRISMA)” statement [13] (Supplementary Table 1). This systematic review was also preregistered at PROSPERO (CRD42024507600).

### Eligibility criteria

Studies meeting the following inclusion criteria were included in this review: (1) a live videoconference or a video recording was used to measure physical fitness, (2) physical fitness tests were used to measure physical fitness, (3) outcome measures of validity, reliability, or feasibility were reported and (4) participants were at least 1 year old. Studies that used a videoconference as a test method or as an intervention method without reviewing the test methodology (e.g. validity, reliability, or feasibility) were excluded. Additionally, studies using apps or other automated data collection were excluded.

### Search

The databases PubMed, EBSCOhost, and Web of Science were used to identify articles. The search was performed in July 2023 and was run from 1966 (or earliest date in the database) to end of June 2023. For all databases we used the following systematic search term strategy. The primary search terms ((“digital”), (“remote”), (“internet-based”), (“mobile applications”), (“apps”), (“mobile apps”), (“video call”), (“video meeting”), (“video-based”), (“videoconferencing”), (“telerehabilitation”)) were each connected (“AND”) separately with the secondary search terms ((“motor skill”), (“motor performance”), (“physical fitness”), (“motor fitness”)) and with the tertiary search terms ((“test”), (“assessment”)). The search was limited to the titles, abstracts, and keywords. No further restrictions were made. An updated literature search was performed in September 2024.

### Study selection

After removing duplicates in Citavi 6.16 (Swiss Academic Software GmbH, Wädenswil, Switzerland) records were screened for title and then abstract. Following this, the full-texts of the relevant studies were screened. The screening process was conducted independently by two researchers (T.K. and A.H.-D.). No automation tool was used in this process. Disagreements concerning the inclusion of full texts were resolved by discussion or by consulting a third reviewer if no consensus was achieved by discussion. For studies excluded in the full-text screening process, reasons for exclusion are noted (Supplementary Table 2). A snowball search was conducted in February 2024 to find further relevant titles from the reference lists and citations of the included studies. Furthermore, systematic reviews were excluded in this review but their reference lists and citations are also screened if they seem relevant to the review question. The updated search was performed by only one reviewer (T.K.).

### Data extraction and synthesis

One reviewer (T.K.) extracted the data from the included studies and another reviewer (A.H.-D.) checked the extracted data to reduce risk of errors. The characteristics of the included studies regarding author and year of publication, country, sample size, age and sex distribution, physical fitness tests, main outcomes of interest for this review (measures of validity, reliability, and feasibility), statistical analyses, and results were extracted. If possible and practical, the individual test results for the subtasks of physical fitness batteries (e.g. the short physical performance battery - SPPB) were reported instead of the total results.

Given the large heterogeneity in the methodologies and results of included studies, a meta-analysis was ruled out. Rather, data were synthesized in summary tables and a narrative synthesis was conducted. Studies were grouped according to the physical fitness components by Caspersen et al. (1985) [7], that can be assigned to the used physical fitness tests (tests can be assigned to multiple components). The fitness components of the tests were determined based on the information contained in the included studies and the listed references. Subgrouping was made based on the used physical fitness tests (e.g. 30s-Sit-to stand test). A description of each study population, the physical fitness tests used, the main outcome of interest, and results is presented. Additionally, an overview of the physical fitness component(s) that can be assigned to the physical fitness tests used in the included studies is presented. In the summary and synthesis, all studies were included. A standardized metric or transformation method was not imposed as the included data were too heterogeneous. The different validity, reliability and feasibility results were summarized by using arbitrary categories and then grouped by the assigned physical fitness components of the physical fitness tests. Categories for validity measures were Good $(r>0.7; \alpha^{\mathrm{a}}>0.8; ICC>0.75; \beta>0.8; PA\geq0.9; rho>0.5)$, Moderate $(r=0.31-0.7; \alpha^{\mathrm{a}}=0.7-0.8; ICC=0.5-0.75; \beta=0.6-0.8; PA=0.8-0.89; rho=0.3-0.5)$ and Poor $(r<0.31; \alpha^{\mathrm{a}}<0.7; ICC<0.5; \beta<0.6; PA<0.8; rho<0.3)$. Categories for reliability measures were Good $(ICC>0.75; \alpha^{\mathrm{b }}>0.8; \kappa>0.6)$, Moderate $(ICC=0.5-0.75; \alpha^{\mathrm{b }}=0.667-0.8; \kappa=0.4-0.6)$ and Poor $(ICC<0.5; \alpha^{\mathrm{b }}<0.667; \kappa<0.4).$ Categories for the feasibility measures were Good (80–100% completion rate), Moderate (60–80% completion rate) and Poor (<60% completion rate). In view of the range of measures and their heterogeneity, we did not evaluate the certainty of evidence.

### Study quality assessment

Study quality was assessed in two parts. For all studies, excluding randomized controlled trials (RCTs), the EPHPP Quality Assessment Tool for Quantitative Studies [14] was used. This tool contains in total 20 items spread over the components selection bias, study design, confounders, blinding, data collection methods, withdrawals and drop-outs, intervention integrity and analyses. With the exceptions of intervention integrity and analyses, all components are rated either strong, moderate or weak. These ratings will be used as a guide for the global study risk of bias rating. The quality assessment was carried out independently by two reviewers (T.K. & A.H.-D.) and disagreements were resolved by discussion.

To assess the methodological quality of the included RCTs we used the Evidence Project’s risk of bias tool [15]. This tool contains eight items, evaluated using the options: no, yes, not applicable, or not reported. The eight items include: (1) Cohort, (2) Control or comparison group, (3) Pre/post intervention data, (4) Random assignment of participants to the intervention, (5) Random selection of participants for assessment, (6) Follow-up rate of 80% or more, (7) Comparison groups equivalent on sociodemographics, and (8) Comparison groups equivalent at baseline on outcome measures. These items can in turn be grouped into three categories: (1) Study design (items 1–3), (2) Participant representativeness (items 4–6), and (3) Equivalence of comparison groups (items 7 & 8). The quality assessment for the RCTs was carried out independently by two reviewers (T.K. & C.N.) as well and disagreements were resolved by discussion.

## Results

**Fig. 1:**
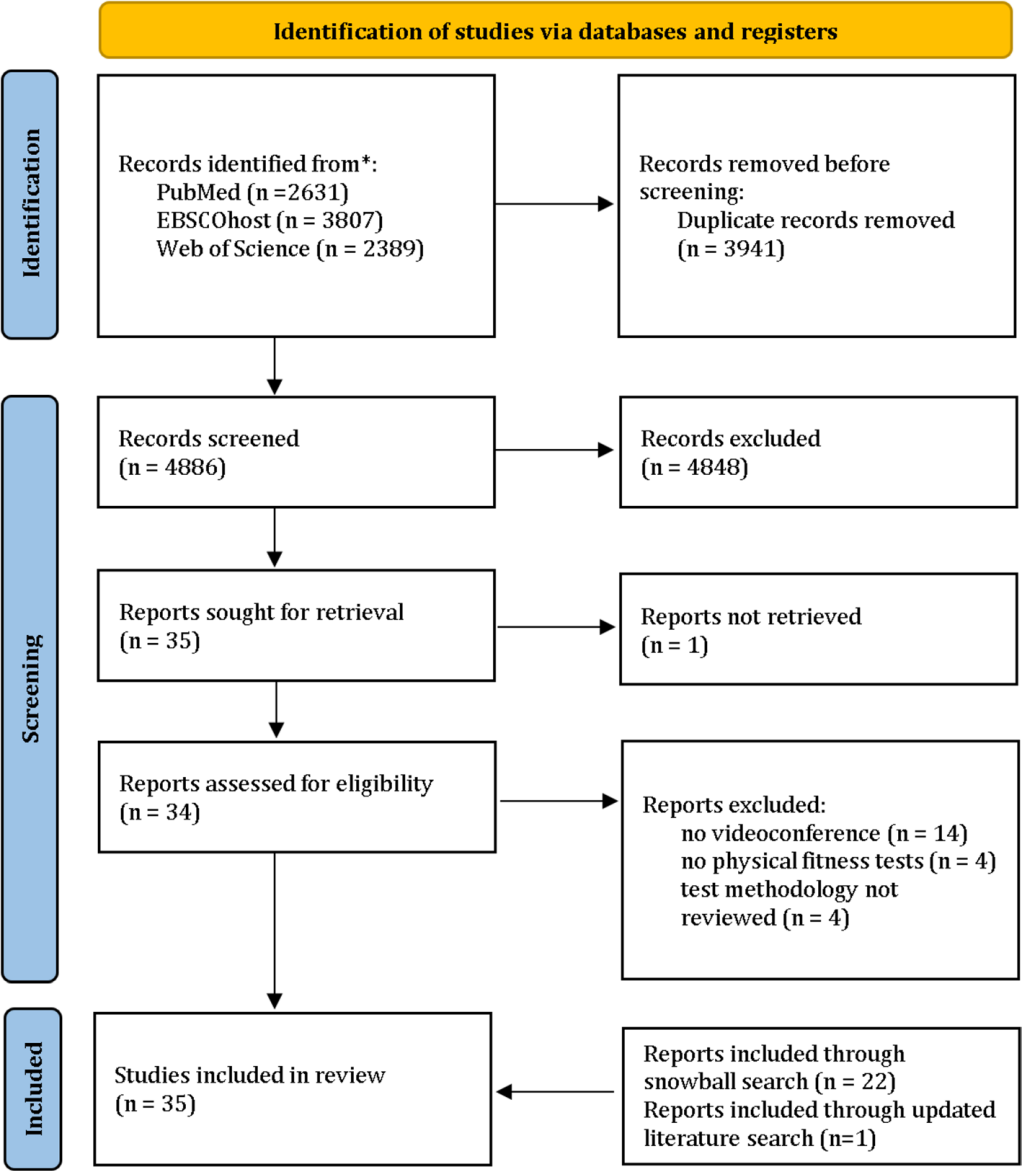
Flow diagram of the screening process (adapted from Page et al., 2021 [13])

The initial search (see Fig. 1) resulted in 8827 publications (2631 publications from PubMed, 3807 publications from EBSCOhost, and 2389 publications from Web of Science). After removing duplicates, 4886 articles remained for the title and abstract screening. After both screening stages, 4851 studies were excluded, and 35 studies remained. After the eligibility screening of the full texts, another 23 articles were excluded. Furthermore, the reference lists and citations screening of all included studies resulted in 22 additional eligible publications. The updated literature search resulted in one additional eligible publication. In total, 35 studies were included in this review.

### Study characteristics

**Fig. 2:**
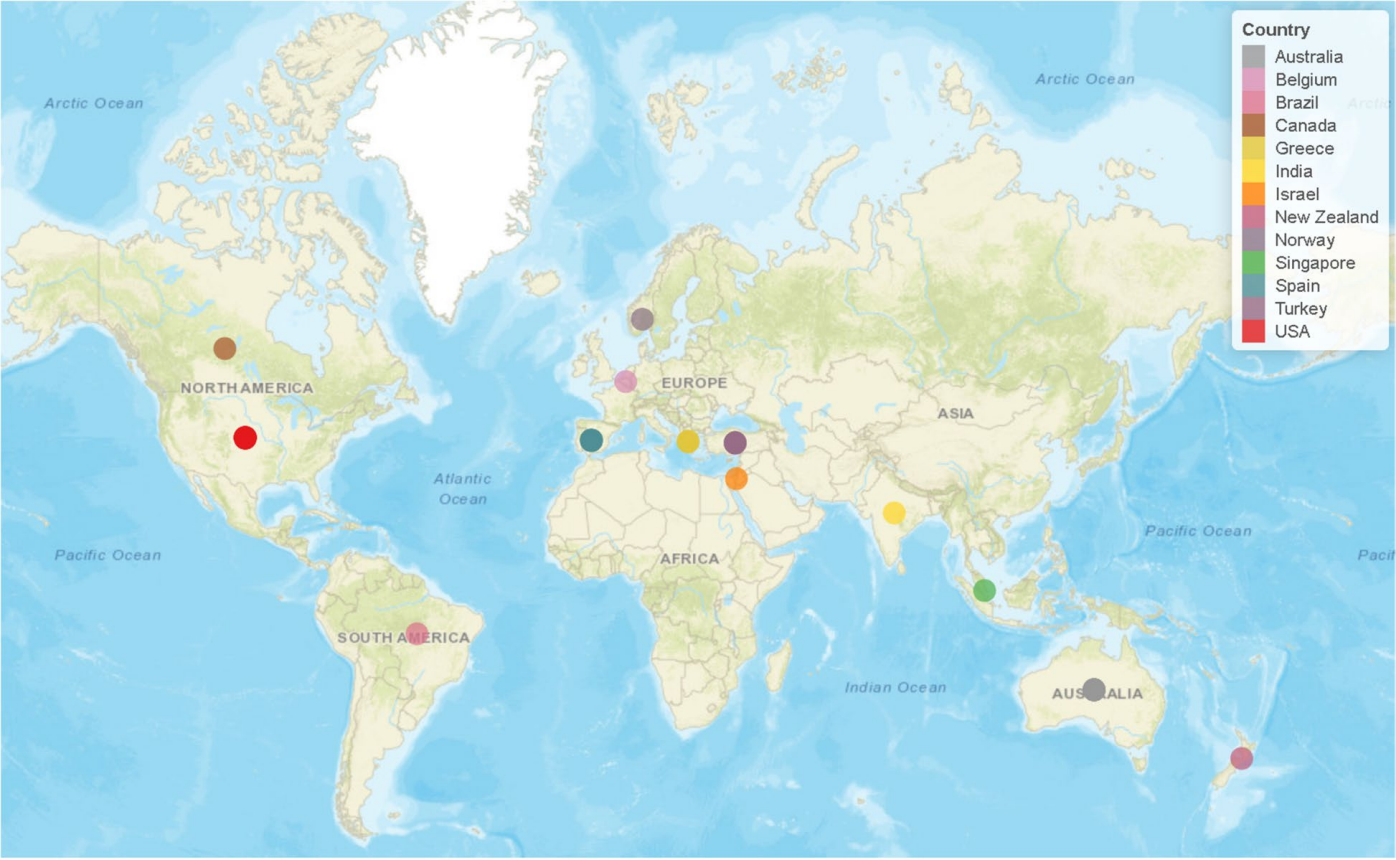
World map displaying the geographical distribution of the included studies (created with EviAtlas; Haddaway et al., 2019 [50])

A summary of study characteristics of the included studies is shown in Table 1. Of the 35 studies, eleven were conducted in Europe (three in Turkey), eleven in North America, nine in Australia-Oceania, three in Asia, and one in South America. In total the included studies were spread across 13 countries which geographical distribution is shown in Fig. 2 (USA=10; Australia=8; Spain=4; Turkey=3; Greece=2; Belgium=1; Brazil=1; Canada=1; India=1; Israel=1; New Zealand=1; Norway=1; Singapore=1). Publication years show that 27 of the included studies were published since 2020. The sample sizes ranged from 10 to 157 participants, and the mean age ranged from 1 to >80 years. Only four studies investigated children and adolescents below the age of 18 years, while 17 studies investigated people 60 years and older. Of the 35 studies, 23 investigated people with different kinds of health conditions, with cancer (4) and diabetes mellitus type 2 (3) being the two most common. On the other hand, twelve studies investigated people without any diagnosed conditions while two of those investigated a mixed sample of people with and without health conditions.

**Table 1:** Details of included studies. ^a^Age range and/or mean age (and standard deviation) as reported; ETUG = Expanded timed up and go; 3M-ST = 3-minute step test; SoT = Sorensen test; 360-TT = 360$^{\circ }$ Turn test; BBS = Berg balance scale; FR = Functional reach test; LR = Lateral reach test; ST = Step test; TUG = Timed up and go; 6M-WT = 6-minute walk test; FN = Finger-nose test; FT = Finger-tapping test; MABC2 = Movement Assessment Battery for Children - Second Edition; CR = Coin rotation task; POMA-G = Tinetti Performance-Oriented Mobility Assessment Gait Scale; GMFM-88 = Gross motor function measure-88; 2M-ST = 2-minute step test; 30s-STS = 30 second sit-to-stand test; 30s-AC = 30-second arm curl test; 1M-PU = 1-minute push-up test; 1M-SU = 1-minute sit-up test; V-SR = V-sit and reach test; WS = Wall sit test; 5XSTS = 5-times sit-to-stand test; KPU = Kneeling push-up test; SITFE = Shirado-Ito trunk flexor endurance test; SB = Standing balance; 4m-WT = 4-meter walk test; CU = Curl-up test; LB = Lateral bridge test; MPU = Modified push-up test; PT = Plank test; 5m-FW = 5-meter fast-paced walk; CRT = Calf raise test; SCT = Stair climb test; SLS = Single leg stance; DGI = Dynamic gait index; FGA = Functional gait assessment; 10XSTS = 10-times sit-to-stand test; UB = Unipedal balance test; 9-PB = 9-hole pegboard test; S-TUG = Supine-timed up and go; SLJ = Standing long jump; 1M-STS = 1-minute sit-to-stand test; CST = Chester step test; SRT = Sitting and rising test; GIFT = Gilboa functional test; SAR = Stand and reach; ICC = Intraclass correlation;* r *= Pearson´s correlation coefficient; $\alpha ^{\textrm{a}}$ = Cronach´s alpha; $\beta$ = Beta regression coefficient; PA = Percentage agreement; IPAQ = International physical activity questionnaire; rho = Spearman correlation coefficient; $\kappa$ = Cohen´s kappa; $\alpha ^{\textrm{b}}$ = Krippendorf´s alpha; DCDQ´07/LDCDQ = Developmental coordination disorder questionnaire 2007/Little developmental coordination disorder questionnaire; DPSQ = Drawing proficiency screening questionnaire

Study	Country	Sample (% female)	Age^a^	Health characteristic	Physical fitness tests	Study quality	Outcomes of interest	Results
Botolfsen et al., 2008 [16]	Norway	28 (82.14)	80.0 (4.14)	Home-dwelling older adults with impaired mobility	ETUG	Moderate	Validity (remote vs. face-to-face)	High (r = 0.85)
							Interrater reliability (remote vs. remote)	ETUG total time and subtasks: Moderate to excellent (ICC = 0.55–0.96)
							Intrarater reliability (remote vs. remote)	ETUG total time and subtasks: Good to excellent (ICC = 0.75–0.97)
							Test-retest reliability (remote vs. remote)	ETUG total time and subtasks: Moderate to good (ICC = 0.54–0.85)
Cox et al., 2013 [17]	Australia	10 (50)	32 (7)	Cystic fibrosis	3M-ST	Strong	Feasibility (remote)	100% completion rate; 90% indicated no preference for in-person versus remote assessment
Palacín-Marín et al., 2013 [18]	Spain	15 (60)	37	Low back pain	SoT	Strong	Criterion validity (remote vs. face-to-face)	Acceptable ($\alpha ^{\textrm{a}}$= 0.796)
							Interrater reliability (remote vs. face-to-face)	Excellent (ICC = 0.92;$\alpha ^{\textrm{a}}$= 0.93)
							Intrarater reliability (remote vs. remote)	Excellent (ICC = 0.94;$\alpha ^{\textrm{a}}$= 0.95)
Russell et al., 2013 [19]	Australia	12 (50)	45–76; 66.1 (8.5)	Parkinson disease	360-TT; BBS; FR; LR; ST; TUG	Weak	Interrater reliability (remote vs. face-to-face)	Overall excellent (ICC$\ge$0.96)
							Intrarater reliability (remote vs. remote)	Overall excellent (ICC$\ge$0.98)
Hwang et al., 2017 [20]	Australia	17 (12); 69 (12)	39–87;	Stable chronic heart failure	6M-WT; TUG	Moderate	Concurrent validity (remote vs. face-to-face)	No difference between remote and face-to-face assessment (p> 0.05)
								6M-WT: Good (ICC = 0.90)
								TUG: Good (ICC = 0.85)
							Interrater reliability (remote vs. face-to-face)	6M-WT: Excellent (ICC> 0.99)
								TUG: Excellent (ICC = 0.95)
							Intrarater reliability (remote vs. remote)	6M-WT: Excellent (ICC > 0.99)
								TUG: Excellent (ICC = 0.96)
Hoenig et al., 2018 [21]	USA	50 (20)	61.3 (1.8)	Veterans with impaired fine/gross motor coordination	FN; FT	Moderate	Criterion validity (remote vs. face-to-face)	FN: Excellent ($\beta$= 0.97–1.00)
								FT: Poor to excellent ($\beta$= 0.35–0.94)
							Interrater reliability (remote vs. remote)	FN: Good to excellent (ICC = 0.88–0.99)
								FT: Moderate to excellent (ICC = 0.59–0.99)
Nicola et al., 2018 [22]	Australia	59 (47)	5–11	School children without any diagnoses	MABC2	Moderate	Concurrent validity (remote vs. face-to-face)	Unacceptable to high level of agreement (PA = 31.67–100%); No difference between remote and face-to-face assessment (p = 0.87)
							Feasibility (remote)	100% completon rate
Cabrera-Martos et al., 2019 [23]	Spain	21 (44.7)	70.9 (9.6)	Parkinson disease	CR; FT	Strong	Interrater reliability (remote vs. face-to-face)	CR: Good to excellent (ICC = 0.89–0.91)
								FT: Excellent (ICC = 0.99–1.00)
Venkataraman et al., 2020 [24]	USA	42 (19)	60.79 (12.25)	Veterans with impaired mobility	POMA-G	Moderate	Criterion validity (remote vs. face-to-face)	Moderate ($\beta$= 0.62–0.80)
							Interrater reliability (remote vs. face-to-face)	Moderate (ICC = 0.66–0.77)
Gavazzi et al., 2021 [25]	USA	21 (57.1)	1–52; 10.1 (11.0)	Leukodys-trophy	GMFM-88	Moderate	Interrater reliability (remote vs. remote)	Excellent (ICC = 0.996)
							Intrarater reliability (remote vs. remote)	Excellent (ICC = 0.999)
Ogawa et al., 2021 [26]	USA	55 (14.6)	74.6 (8.1)	Community-dwelling veterans	2M-ST; 30s-STS; 30s-AC	Strong	Interrater reliability (remote vs. remote)	2M-ST: Excellent (ICC = 0.999)
								30s-STS: Excellent (ICC = 0.989)
								30s-AC: Excellent (ICC = 0.992)
Bhagat et al., 2022 [27]	India	100 (39)	43.75 (11.31)	Diabetes mellitus type 2	1M-PU; 1M-SU; V-SR ;WS	Poor	Feasibility (remote)	100% completion rate; no safety issues
Bowman et al., 2022 [28]	Australia	30 (41)	62.5	Cancer (various forms)	30s-STS	Moderate	Convergent validity (remote vs. remote)	Moderate association with physical activity IPAQ (rho = 0.46 ($p<0.01$))
							Discriminant validity (remote vs. remote)	No association with perceived exertion (rho = −0.12 (<0.53))
							Feasibility (remote)	94% completion rate; no safety issues
Espin et al., 2022 [29]	Spain	96 (50)	18–65	Healthy adults	5XSTS; KPU; SITFE	Moderate	Interrater Reliability (remote vs. remote)	5XSTS: Excellent (ICC = 0.99)
								KPU: Excellent (ICC = 0.96)
								SITFE: Excellent (ICC = 0.97)
							Test-retest reliability (remote vs. remote)	5XSTS: Excellent (ICC = 0.92–0.98)
								KPU: Excellent (ICC = 0.96–0.98)
								SITFE: Excellent (ICC = 0.93)
							Feasibility (remote)	100% completion rate; short test duration; excellent feasibility score (4.5–4.7 of 5)
Fyfe et al., 2022 [30]	Australia	38 (63.15)	69.8 (3.8)	Community-dwelling older adults	5XSTS; 30s-STS; SB	Strong	Feasibility (remote)	100% completion rate
Guidarelli et al., 2022 [31]	USA	118 (28.25)	62.5 (11.5)	Breast or prostate cancer survivors and healthy adults	4m-WT; 5XSTS; SB; TUG	Strong	Interrater reliability (remote vs. remote)	4m-WT: Moderate (ICC = 0.62)
								5XSTS: Moderate (ICC = 0.65)
								SB: Unacceptable ($\alpha ^{\textrm{b}}$= 0.59)
								TUG: Excellent (ICC = 0.98)
							Intrarater reliability (remote vs. remote)	4m-WT: Good (ICC = 0.87)
								5XSTS: Excellent (ICC = 0.92)
								SB: Nearly perfect ($\kappa$= 0.82)
								TUG: Excellent (ICC = 0.96)
Güngör et al., 2022 [32]	Turkey	80 (65)	18–40; 26.18 (4.83)	Healthy adults	30s-STS; CU; FR; LB; MPU; PT; TUG	Moderate	Validity (remote vs. face-to-face)	30s-STS: High (r = 0.92)
								CU: High (r = 0.93)
								FR: High (r = 0.96)
								LB: High (r = 0.92–0.94)
								MPU: High (r = 0.91)
								PT: High (r = 0.93)
								TUG: High (r = 0.94)
							Test-retest reliability (remote vs. remote)	30s-STS: Excellent (ICC = 0.95)
								CU: Excellent (ICC = 0.96)
								FR: Excellent (ICC = 0.97)
								LB: Excellent (ICC = 0.91–0.93)
								MPU: Excellent (ICC = 0.94)
								PT: Excellent (ICC = 0.97)
								TUG: Excellent (ICC = 0.97)
							Feasibility (remote)	100% completion rate
Lawford et al., 2024 [33]	Australia	57 (70)	63.1 (9.3)	Chronic lower limb musculoskeletal pain	5m-FW; 30s-STS; CRT; SCT; SLS; ST; TUG	Strong	Test-retest reliability (remote vs. remote)	5m-FW: Moderate (ICC = 0.71)
								30s-STS: Good (ICC = 0.77)
								CRT: Good (ICC = 0.84–0.85)
								SCT: Excellent (ICC = 0.91)
								SLS: Good (ICC = 0.69–0.84)
								ST: Good (ICC = 0.79–0.81)
								TUG: Good (ICC = 0.86)
Pelicioni et al., 2022 [34]	New Zealand	15 (53.33)	64–78; 71.7	Healthy older adults	BBS; DGI; FGA; TUG	Moderate	Criterion validity (remote vs. face-to-face)	Live telehealth :
								BBS : Moderate (r = −0.52 ($p < 0.05$))
								DGI : Moderate (r = −0.53 ($p < 0.05$))
								FGA : Moderate (r = −0.68 ($p < 0.05$))
								TUG : Moderate (r = −0.55–0.64 ($p < 0.05$))
								Recorded telehealth :
								BBS : Moderate (r = −0.56 ($p < 0.05$))
								DGI : Moderate (r = −0.69 ($p < 0.05$))
								FGA : Moderate (r = −0.69 ($p < 0.05$))
								TUG : Moderate to High (r = −0.64–0.71 ($p < 0.05$))
							Interrater reliability (remote vs. remote)	BBS : Excellent (ICC = 0.96)
								DGI : Good (ICC = 0.85)
								FGA : Good (ICC = 0.80)
								TUG : Excellent (ICC = 1.00)
							Intrarater reliability (remote vs. remote)	BBS : Good (ICC = 0.78–0.82)
								DGI : Good (ICC = 0.86–0.88)
								FGA : Good (ICC = 0.87)
								TUG : Good (ICC = 0.79–0.85)
Peyrusqué et al., 2022 [35]	Canada	15 (60)	69.3 (3.6)	Healthy older adults	4m-WT; 5XSTS; 10XSTS; 30s-STS; TUG; UB	Moderate	Relative reliability (remote vs. face-to-face)	4m-WT: Moderate to good (ICC = 0.62–0.77)
								5XSTS: Excellent (ICC = 0.96)
								10XSTS: Excellent (ICC = 0.99)
								30s-STS: Excellent (ICC = 0.97)
								TUG: Good to Excellent (ICC = 0.83–0.93)
								UB: Good (ICC = 0.79)
Aktan et al., 2023 [36]	Turkey	50 (38)	54.5 (6.3)	Diabetes mellitus type 2	30s-STS	Strong	Interrater reliability (remote vs. face-to-face)	30s-STS: Excellent (ICC =0.93)
Button et al., 2023 [37]	USA	15 (43)	3.4 (0.5)	Heathy preschool children	9-PB; S-TUG; SLJ; UB	Moderate	Validity (remote vs. face-to-face)	No statistical differences between remote and face-to-face measures (p = 0.36–0.90)
								9-PB: Small (r = −0.75- −0.151)
								S-TUG: Medium (r = 0.485)
								SLJ: High (r = 0.619)
								UB: Medium to high (r = 0.375–0.740)
Hoge et al., 2023 [38]	USA	30 (93.3)	46.2 (11.9)	Systemic lupus erythematosus	4m-WT; 5XSTS; SB	Moderate	Test-retest reliability (remote vs. face-to-face)	4m-WT: Poor (ICC = 0.23–0.48)
								5XSTS: Moderate (ICC = 0.66)
								SB: Excellent (ICC = 0.91)
Mavronasou et al., 2024 [39]	Greece	25 (40)	53 (10)	Post-COVID-19 symptoms	1M-STS; 4m-WT; 5XSTS; CST	Moderate	Interrater reliability (remote vs. face-to-face)	1M-STS: Excellent (ICC = 0.977)
								4m-WT: Good (ICC = 0.777)
								5XSTS: Good (ICC = 0.792)
								CST: Good (ICC = 0.871)
Mehta et al., 2023 [40]	USA	52 (40.4)	18–61; 28.3 (11.3)	Healthy adults	4m-WT; 30s-STS; ST; TUG	Strong	Interrater reliability (remote vs. face-to-face)	4m-WT: Good (ICC = 0.833)
								30s-STS: Excellent (ICC = 0.947)
								ST: Excellent (ICC = 0.932)
								TUG: Good (ICC = 0.867)
Ng et al., 2023 [41]	Singapore	63 (42.85)	26.1 (7.3)	Healthy adults	1M-STS; 30s-STS	Poor	Feasibility (remote)	100% completion rate
Núñez-Cortés et al., 2023 [42]	Spain	79 (86.10)	24–52;	Long COVID	30s-STS	Moderate	Feasibility (remote)	100% completion rate
Pepera et al., 2023 [43]	Greece	23 (25)	39–85; 61 (13)	Diabetes mellitus type 2	6M-WT	Strong	Validity (remote vs. face-to-face)	High (r = 0.76 ($p < 0.001$))
							Test-retest reliability (remote vs. remote)	Excellent (ICC = 0.98)
Silva et al., 2023 [44]	Brazil	30 (86.7)	69.77 (6.6)	Community-dwelling older adults	5XSTS; 30s-STS; SRT	Moderate	Intrarater reliability (remote vs. remote)	5XSTS: Excellent (ICC = 0.93)
								30s-STS: Excellent (ICC = 0.91–0.98)
								SRT: Good (ICC = 0.90)
Sinvani et al., 2023 [45]	Israel	157 (56.7)	3–7; 4.98 (1.13)	Healthy children	GIFT	Poor	Concurrent validitiy (remote vs. remote)	Low to medium correlation with DCDQ´07/LDCDQ (r = 0.29 ($p < 0.001$))
								Small to medium correlation with DPSQ (r = −0.35 ($p < 0.001$))
							Construct validitiy (remote vs. remote)	Medium to high correlation with age (r = 0.33–0.57 ($p < 0.05$))
								Girls have better performance than boys ($p<0.05$)
		40 (58)	3–7; 5.17 (1.06)				Interrater reliability (remote vs. remote)	Excellent (r = 0.97 ($p < 0.001$))
Steffens et al., 2023 [46]	Australia	37 (64.9)	54.00	Gastrointestinal cancer	5XSTS	Moderate	Interrater reliability (remote vs. face-to-face)	Excellent (ICC = 0.957 ($p < 0.001$))
							Feasibility	100% comletion rate; no safety issues
Buckinx et al., 2024 [47]	Belgium	45 (48.9)	77.7 (7.7)	Healthy older adults	2M-ST; 4m-WT; 5XSTS; 10XSTS; 30s-STS; TUG; SAR; UB	Poor	Interrater reliability (remote vs. remote)	2M-ST: Excellent (ICC = 0.92)
								4m-WT: Excellent (ICC = 0.91–0.98)
								5XSTS: Excellent (ICC = 0.98)
								10XSTS: Excellent (ICC = 0.99)
								30s-STS: Excellent (ICC = 0.95)
								SAR: Excellent (ICC = 1.00)
								TUG: Excellent (ICC = 0.92–0.97)
								UB: Excellent (ICC = 0.98)
							Intrarater reliability (remote vs. face-to-face)	2M-ST: Good (ICC = 0.85)
								4m-WT: Good to excellent (ICC = 0.88–0.96)
								5XSTS: Excellent (ICC = 0.97)
								10XSTS: Excellent (ICC = 0.97)
								30s-STS: Excellent (ICC = 0.93)
								SAR: Excellent (ICC = 1.00)
								TUG: Excellent (ICC = 0.91–0.95)
								UB: Excellent (ICC = 0.93)
Gell et al., 2024 [48]	USA	39 (79)	61–84; 70.4 (5.7)	Cancer survivors	5XSTS; 30s-STS; SB	Strong	Feasibility (remote)	95% completion rate
Lai et al., 2024 [11]	USA	19 (44)	Cerebral palsy 17.4 (1.9)	Healthy and cerebral palsy	5XSTS; 6M-WT; TUG	Strong	Convergent validity (remote vs. face-to-face)	5XSTS: Excellent (ICC = 0.95 (p = 0.01))
								6M-WT: Good (ICC = 0.83 (p = 0.18))
								6M-WT: High (r = 0.83 ($p < 0.001$))
								TUG: Excellent (ICC = 0.92 (p = 0.01)
		10 (50)	Healthy 19.3 (1.2)				Interrater reliability (remote vs remote)	5XSTS: Excellent (ICC = 0.998 ($p < 0.001$))
								6M-WT: Excellent (ICC = 0.999 ($p < 0.001$))
								TUG: Excellent (ICC = 0.999 ($p < 0.001$))
							Feasibility (remote)	100% completion rate; no safety issues; teleassessment took 20% longer (p = 0.003); people with cerebral palsy needed more time (p = 0.01)
Tütüneken et al., 2024 [49]	Turkey	61 (27.9)	59.11 (10.05)	Stroke	30s-STS; TUG	Strong	Validity (remote vs. face-to-face)	30s-STS: High (r = 0.94 ($p < 0.001$))
								TUG: High (r = 0.97 ($p < 0.001$))
							Interrater reliability (remote vs. remote)	30s-STS: Good ($\alpha ^{\textrm{b}}$= 0.981)
								TUG: Good ($\alpha ^{\textrm{b}}$= 0.996)
							Test-retest reliability (remote vs. remote)	30s-STS: Excellent (ICC = 0.992)
								TUG: Excellent (ICC = 0.998)

### Study qualities

The results of the quality assessments are shown in Table 2 (non-RCTs) and Table 3 (RCTs). Of the non-RCTs, 10 studies were rated with a strong quality rating, 17 studies were rated with a moderate quality rating, and five studies were rated with a weak quality rating. For the component of the selection bias most studies had a moderate rating while no study had a strong rating. In the study design component all studies had a moderate rating. In terms of blinding most studies had a weak rating and no study had a strong rating. For the components of confounders and data collection methods all studies had strong ratings without exception. In the withdrawals and drop-outs all component all studies but two had strong ratings. The three RCTs showed primarily strong ratings in the study design category, whereas one study did not use both an control group and an pre/post design. Regarding participant representativeness the RCTs showed almost without exception strong ratings, while only one study did not randomly select the participants for the assessment. In terms of the equivalence of comparison groups a mixed picture is seen. One study showed strong ratings throughout, while the other two studies did either not report on all items or the items were not applicable.

**Table 2:** Study quality assessment (EPHPP Quality Assessment Tool for Quantitative Studies)

Author & year	Selection bias	Study design	Confounders	Blinding	Data collection methods	Withdrawals and drop-outs	Global rating
Botolfsen et al., 2008 [16]	Moderate	Moderate	Strong	Weak	Strong	Strong	Moderate
Cox et al., 2013 [17]	Moderate	Moderate	Strong	Moderate	Strong	Strong	Strong
Palacín-Marín et al., 2013 [18]	Moderate	Moderate	Strong	Moderate	Strong	Strong	Strong
Russell et al., 2013 [19]	Weak	Moderate	Strong	Weak	Strong	Strong	Weak
Hwang et al., 2017 [20]	Moderate	Moderate	Strong	Weak	Strong	Strong	Moderate
Hoenig et al., 2018 [21]	Moderate	Moderate	Strong	Weak	Strong	Strong	Moderate
Nicola et al., 2018 [22]	Weak	Moderate	Strong	Moderate	Strong	Strong	Moderate
Cabrera-Martos et al., 2019 [23]	Moderate	Moderate	Strong	Moderate	Strong	Moderate	Strong
Venkataraman et al., 2020 [24]	Weak	Moderate	Strong	Moderate	Strong	Strong	Moderate
Gavazzi et al., 2021 [25]	Moderate	Moderate	Strong	Weak	Strong	Strong	Moderate
Ogawa et al., 2021 [16]	Moderate	Moderate	Strong	Moderate	Strong	Strong	Strong
Bhagat et al., 2022 [27]	Weak	Moderate	Strong	Weak	Strong	Strong	Weak
Bowman et al., 2022 [28]	Moderate	Moderate	Strong	Weak	Strong	Strong	Moderate
Espin et al., 2022 [29]	Weak	Moderate	Strong	Moderate	Strong	Strong	Moderate
Güngör et al., 2022 [32]	Moderate	Moderate	Strong	Weak	Strong	Strong	Moderate
Lawford et al., 2024 [33]	Moderate	Moderate	Strong	Moderate	Strong	Strong	Strong
Pelicioni et al., 2022 [34]	Moderate	Moderate	Strong	Weak	Strong	Strong	Moderate
Peyrusqué et al., 2022 [35]	Moderate	Moderate	Strong	Weak	Strong	Strong	Moderate
Aktan et al., 2023 [36]	Moderate	Moderate	Strong	Moderate	Strong	Strong	Strong
Button et al., 2023 [37]	Moderate	Moderate	Strong	Weak	Strong	Strong	Moderate
Hoge et al., 2023 [38]	Moderate	Moderate	Strong	Weak	Strong	Strong	Moderate
Mavronasou et al., 2024 [39]	Moderate	Moderate	Strong	Weak	Strong	Strong	Moderate
Mehta et al., 2023 [40]	Moderate	Moderate	Strong	Moderate	Strong	Strong	Strong
Ng et al., 2023 [41]	Weak	Moderate	Strong	Weak	Strong	Strong	Weak
Núñez-Cortés et al., 2023 [42]	Moderate	Moderate	Strong	Weak	Strong	Strong	Moderate
Pepera et al., 2023 [43]	Moderate	Moderate	Strong	Moderate	Strong	Strong	Strong
Silva et al., 2023 [44]	Moderate	Moderate	Strong	Weak	Strong	Strong	Moderate
Sinvani et al., 2023 [45]	Weak	Moderate	Strong	Weak	Strong	Strong	Weak
Steffens et al., 2023 [46]	Moderate	Moderate	Strong	Weak	Strong	Strong	Moderate
Buckinx et al., 2024 [47]	Weak	Moderate	Strong	Weak	Strong	Weak	Weak
Lai et al., 2024 [11]	Moderate	Moderate	Strong	Moderate	Strong	Strong	Strong
Tütüneken et al., 2024 [49]	Moderate	Moderate	Strong	Moderate	Strong	Strong	Strong

**Table 3:** Study quality assessment of the included RCT’s (Evidence Project risk of bias tool)

	Study design			Participant representativeness			Equivalence of comparison groups	
Author & year	Cohort	Control or comparison group	Pre/post intervention data	Random assignment of participants to the intervention	Random selection of participants for assessment	Follow-up rate of 80% or more	Comparison groups equivalent on sociodemographics	Comparison groups equivalent at baseline on outcome measures
Fyfe et al., 2022 [30]	Yes	Yes	Yes	Yes	Yes	Yes	Yes	NR
Gell et al., 2022 [48]	Yes	Yes	Yes	Yes	No	Yes	Yes	Yes
Guidarelli et al., 2022 [31]	Yes	No	No	Yes	Yes	Yes	NA	NR

### Summary of validity, reliability and feasibility results

A summary of the validity, reliability, and feasibility grouped by the physical fitness components of the physical fitness tests is shown in Table 4. Out of the 11 physical fitness components, balance (108) contains overall the most measures, followed by muscular strength (91), muscular endurance (43), power (23), coordination (21), cardiorespiratory endurance (14), speed (11), flexibility (4), and agility (1). No measures were recorded for body composition and reaction time. Regarding validity, balance contained for the most part good measures (12/25), as well as muscular strength (13/17), muscular endurance (5/8), power (3/5), and cardiorespiratory endurance (4/4). Coordination contained four good measures (out of 10), agility one (out of one), and flexibility contained no validity measures. Regarding reliability, all components predominantly contained good measures (balance (63/69), muscular strength (51/55), muscular endurance (22/22), power (13/13), speed (7/11), cardiorespiratory endurance (8/8), coordination (7/8) and flexibility (3/3)). Regarding feasibility, all components solely contained good measures (muscular strength (19/19), balance (14/14), muscular endurance (13/13), power (5/5), cardiorespiratory endurance (2/2), flexibility (1/1) and coordination (1/1)).

**Table 4:** Summary table for the validity, reliability and feasibility measures of the physical fitness tests grouped by physical fitness component

	Physical fitness component										
	Cardiorespiratory endurance	Muscular endurance	Muscular strength	Body composition	Flexibility	Agility	Balance	Coordina-tion	Speed	Power	Reaction time
Number of measures on validity	4	8	17	0	0	1	25	10	0	5	0
Good	4	5	13	0	0	0	12	4	0	3	0
Moderate	0	2	3	0	0	1	12	5	0	1	0
Poor	0	1	1	0	0	0	1	3	0	1	0
Number of measures on reliability	8	22	55	0	3	0	69	8	11	13	0
Good	8	22	51	0	3	0	63	7	7	13	0
Moderate	0	0	4	0	0	0	5	1	3	0	0
Poor	0	0	0	0	0	0	1	0	1	0	0
Number of measures on feasibility	2	13	19	0	1	0	14	1	0	5	0
Good	2	13	19	0	1	0	14	1	0	5	0
Moderate	0	0	0	0	0	0	0	0	0	0	0
Poor	0	0	0	0	0	0	0	0	0	0	0

### Physical fitness components

In total, 48 different physical fitness tests were used in the included studies. These can be assigned to nine of the eleven physical fitness components by Caspersen et al. (1985) [7], with body composition and reaction time containing no physical fitness tests. Out of the 48 physical fitness tests, 13 tests were used in more than one study and the remaining 35 tests were used once. 29 of the physical fitness tests can be assigned to one physical fitness components, 18 tests can be assigned to two components, and one test can be assigned to four components. A tabular overview of the 48 physical fitness tests and their respective physical fitness component(s) is shown in Supplementary Table 3.

#### Balance

Of the 48 physical fitness tests, 17 can be used to assess balance. Among them, the *30-second sit-to-stand test *(*30s-STS*) was the most frequently used, featured in 14 studies. It demonstrated a high validity correlation for comparing remote to face-to-face (R2F) assessments [49], as well as a high [31] and moderate [28] correlation for comparing remote-to-remote (R2R) assessments. Regarding reliability, the *30s-STS* consistently showed good to excellent correlations for interrater reliability (R2F [36, 40]; R2R [26, 47, 49]), intrarater reliability (R2F [47]; R2R [44]), test-retest reliability (R2R [32, 33, 49]), and relative reliability (R2F [35]). Feasibility results indicated completion rates over 94% [28, 30, 32, 41, 42, 48]. The *5-times sit-to-stand test *(*5XSTS*) was used in 11 studies and showed an excellent validity correlation for the R2F condition [11]. For reliability, results indicated good to excellent (R2F [39, 46]; R2R [11, 29, 47]) as well as moderate (R2R [31]) correlations for interrater reliability, consistently excellent correlations for intrarater reliability (R2F [47]; R2R [31, 44]), an excellent (R2R [29]) and a moderate (R2F [38]) correlation for test-retest reliability, and an excellent correlation for relative reliability (R2F [35]). Feasibility outcomes showed completion rates over 95% [11, 29, 30, 46, 48]. The *Timed up and go test *(*TUG*) was also featured in 11 studies, showing good to excellent/high validity correlations for the R2F condition [11, 20, 49] as well as a high [32] and moderate [34] correlation for the R2R condition. Reliability results consistently demonstrated good to excellent correlations for interrater reliability (R2F [19, 20, 40]; R2R [11, 31, 34, 47, 49]), intrarater reliability (R2F [47]; R2R [19, 20, 31, 34]), test-retest reliability (R2R [32, 33, 49]), and relative reliability (R2F [35]). Feasibility results for the *TUG *showed a 100% completion rate [11, 32]. The *Standing balance test* (*SB*) was utilized in four studies, none of which examined validity measures. Results for the interrater reliability showed an unacceptable score for the R2R condition [31], while the intrarater reliability demonstrated a nearly perfect score for the R2R condition [31], and the test-retest reliability an excellent correlation for the R2F condition [38]. Feasibility outcomes for the *SB* showed completion rates over 95% [30, 48]. The *Unipedal balance test* (*UB*), used in three studies, demonstrated medium to high validity correlations (R2F [37]) as well as excellent correlations for the interrater reliability (R2R [47]), intrarater reliability (R2F [47]), and a good correlation for the relative reliability (R2F [35]). The *Berg balance scale* (*BBS*), included in two studies, displayed moderate validity correlations for the R2F condition [34]. Interrater reliability results consistently showed excellent correlations (R2F [19]; R2R [34]), and intrarater reliability displayed good to excellent correlations (R2R [19, 34]). The *Functional reach test* (*FR*), used in two studies, demonstrated a high validity correlation for the R2R condition [32]. Reliability results consistently showed excellent correlations for interrater reliability (R2F [19]), intrarater reliability (R2R [19]), and test-retest reliability (R2R [32]). Feasibility results indicated a 100% completion rate [32]. The $360^{\circ }$ *Turn test *(*360-TT*), used in one study [19], showed excellent correlations for both interrater reliability (R2F) and intrarater reliability (R2R). The *Dynamic gait index* (*DGI*), evaluated in one study [34], demonstrated a moderate validity correlation (R2R) and good correlations for both interrater and intrarater reliability. The *Expanded timed up and go test* (*ETUG*), featured in one study [16], exhibited a high validity correlation (R2F), moderate to excellent correlations for interrater reliability (R2R), good to excellent correlations for intrarater reliability (R2R), and moderate to good correlations for test-retest reliability (R2R). The *Functional gait assessment test *(FGA), used in one study [34], showed a moderate validity correlation (R2R) and good correlations for both interrater and intrarater reliability (R2R). The *Lateral reach test* (*LR*), included in one study [19], demonstrated excellent correlations for both interrater reliability (R2F) and intrarater reliability (R2R). The *Movement Assessment Battery for Children - Second Edition *(*MABC2*), assessed in one study [22], showed unacceptable to high levels of percentage agreement for validity (R2F) and a 100% completion rate. The *Tinetti Performance-Oriented Mobility Assessment Gait Scale *(*POMA-G*), used in one study [24], exhibited a moderate validity coefficient and a moderate coefficient for interrater reliability (R2F). The *Single leg stance* (*SLS*), featured in one study [33], demonstrated a good correlation for test-retest reliability (R2R). The *Sitting and rising test *(*SRT*), used in one study [44], showed a good correlation for interrater reliability (R2R). The *Step test* (*ST*), included in one study [19], demonstrated excellent correlations for both interrater reliability (R2F) and intrarater reliability (R2R).

#### Muscular strength

Of the 48 physical fitness tests, 16 can be used to assess muscular strength. The three most frequently used tests for this purpose were the *30s-STS*, the *TUG*, and the *5XSTS* (for results, see subsection Balance). The *1-minute sit-to-stand test* (*1M-STS*) , featured in two studies, demonstrated an excellent correlation for the interrater reliability in the R2F condition [39], and a 100% completion rate [41]. The *10-times sit-to-stand test* (*10XSTS*) , also used in two studies, showed excellent correlations for interrater reliability (R2R [47]), intrarater reliability (R2F [47]), and relative reliability (R2F [35]). The *1-minute push-up test* (*1M-PU*) was used in one study [27], which reported a 100% completion rate for feasibility. Similarly, the *1-minute sit-up test* (*1M-SU*), assessed in the same study [27], also reported a 100% completion rate for feasibility. The *30-second arm curl test* (*30s-AC*) was featured in one study [26], which found an excellent correlation for interrater reliability (R2R). The *Calf raise test* (*CRT* ), used in one study [33], showed a good correlation for test-retest reliability (R2R). The *Curl-up test* (*CU*), included in one study [32], demonstrated a high validity correlation and an excellent correlation for test-retest reliability (R2R), with a 100% completion rate. The *Kneeling push-up test* (*KPU*), used in one study [29], demonstrated excellent correlations for both interrater and test-retest reliability (R2R). Additionally, feasibility results showed a 100% completion rate [29]. The *Lateral bridge test* (*LB*), assessed in one study [32], exhibited a high validity correlation, an excellent test-retest reliability correlation (R2R), and a 100% completion rate for feasibility. The *Modified push-up test *(*MPU*), used in one study [32], showed high validity and excellent test-retest reliability (R2R), along with a 100% completion rate for feasibility. Similarly, the *Plank Test* (*PT*), included in the same study [32], showed high validity and excellent test-retest reliability (R2R), with a 100% completion rate for feasibility. The *Wall sit test* (*WS*), used in one study [27], exhibited a 100% completion rate. Additionally, the *ETUG* can also be used to assess muscular strength (for results, see subsection Balance).

#### Muscular endurance

Out of the 48 physical fitness tests, 12 can be used to assess muscular endurance. The most frequently used test for this purpose was the *30s-STS* (for results, see subsection Balance). The *Shirado-Ito trunk flexor endurance test* (*SITFE*), featured in one study [29], showed excellent correlations for interrater and test-retest reliability (R2R). Additionally, feasibility results indicated a 100% completion rate [29]. The *Sorensen test* (*SoT*), also used in one study [18], showed acceptable validity and excellent correlations for interrater (R2F) and intrarater (R2R) reliability. Moreover, the *1M-STS, 1M-PU, 1M-SU, 30s-AC, CRT, CU, KPU, MPU* and the *PT* (for results, see subsection Muscular strength) can be used to assess muscular endurance.

#### Coordination

Of the 48 physical fitness tests, eight can be used to assess coordination. The *Finger-tapping test* (*FT*) was featured in two studies, which found a good to excellent validity correlation (R2F [21]). For interrater reliability, a moderate to excellent correlation for the R2R condition [21] and an excellent correlation for the R2F condition [23] were found. The *9-hole pegboard test *(*9-PB*), assessed in one study [37], showed a small validity correlation (R2F). For the *Coin rotation task* (*CR*), used in one study [23], a good to excellent interrater reliability correlation (R2F) was found. The *Finger-nose test* (*FN*), included in one study [21], demonstrated an excellent validity result (R2F) and an excellent interrater reliability correlation (R2R). The *Gilboa functional test* (*GIFT*), used in one study [45], showed low to medium and medium to high validity correlations, along with an excellent interrater reliability correlation (R2R). The *Gross motor function measure-88* (*GMFM-88*), assessed in one study [25], exhibited excellent correlations for both interrater and intrarater reliability (R2R). For the *Supine-timed up and go test* (*S-TUG*), used in one study [37], a medium validity correlation (R2F) was found. The *MABC2* can also be used to assess coordination (for results, see subsection Balance).

#### Cardiorespiratory endurance

Out of the 48 physical fitness tests, four can be used to assess cardiorespiratory endurance. The *6-minute walk test *(*6M-WT*), featured in three studies, found good and high validity correlations for the R2F condition [11, 20, 43]. Reliability results showed excellent correlations for interrater reliability (R2F [20]; R2R [11]), intrarater reliability (R2R [20]), and test-retest reliability (R2R [43]). Feasibility outcomes showed a 100% completion rate [11]. The *2-minute step test* (*2M-ST*), assessed in two studies, demonstrated excellent correlations for interrater reliability (R2R [26, 47]) and a good correlation for intrarater reliability (R2F [47]). The *3-minute step test* (*3M-ST*), used in one study [17], exhibited a 100% completion rate. The *Chester step test* (*CST*), featured in one study [39], showed a good interrater reliability correlation (R2F).

#### Flexibility

Of the 48 physical fitness tests, three can be used to assess flexibility. The *Stand and Reach Test* (*SAR*), used in one study [47], showed excellent correlations for both interrater (R2R) and intrarater (R2F) reliability. For the *V-sit and reach test* (V-SR), featured in one study [27], a 100% completion rate was reported. The *SRT* can also be used to assess flexibility (for results, see subsection Balance).

#### Speed

Out of the 48 physical fitness tests, three can be used to assess speed. The most frequently used test for this purpose was the *4-meter walk test* (*4m-WT*). This test was used in six studies, which solely assessed reliability measures. Results for interrater reliability showed good correlations for the R2F condition [39, 40] and both moderate [31] and excellent [47] correlations for the R2R condition. For intrarater reliability, good to excellent correlations were found (R2F [47]; R2R [31]), whereas test-retest reliability showed a poor correlation (R2F [38]), and relative reliability demonstrated moderate to good correlations (R2F [35]). The *5-meter fast-paced walk* (*5m-FW*), featured in one study [33], showed a moderate correlation for test-retest reliability (R2R). For the *Stair Climb Test* (*SCT*), used in one study [33], an excellent correlation for the test-retest reliability (R2R) was found.

#### Power

Of the 48 physical fitness tests three can be used to assess power. The *30s-STS* was the most frequently used test to assess power (for results see subsection Balance) followed by the *10XSTS* (for results see subsection Muscular strength). The *Standing long jump test* (*SLJ*), featured in one study [37], showed a high validity correlation for the R2F condition.

#### Agility

Of the 48 physical fitness tests only the *S-TUG* was used to assess agility (for results see subsection Coordination).

## Discussion

This systematic review examined the evidence for remotely delivered physical fitness tests and their validity, reliability, and feasibility in assessing physical fitness across all age groups. Our results showed that a significant number of physical fitness tests (48) were used remotely. However, only 13 of them were used in more than one study. Additionally, less than half of these physical fitness tests (23) were investigated for their validity, 19 for their feasibility, and 39 for their reliability. These findings suggest that although most physical fitness tests demonstrate good reliability, data on their validity is lacking. This gap in validity data should be carefully considered when using a physical fitness test in a remote setting. This is in line with the findings of the systematic review from Heslop et al. (2023) [12], which highlights a lack of evidence for acceptability, feasibility, and the agreement between face-to-face and remote methods. Furthermore, the physical fitness tests did not cover all eleven physical fitness components. Notably, the components of body composition, and reaction time were not assessed by any remotely delivered physical fitness test, and the assessments were unevenly distributed across the other components. This lack of representation for body composition and reaction time is significant, as these dimensions are essential for understanding broader health risks [51] and physical capabilities. For instance, reaction time plays a crucial role in activities requiring quick decision-making and is a vital component of functional independence and the prevention for the risk of falling, especially in older adults [52, 53]. Our results also showed that most studies (27 of 35) were published since 2020, highlighting the impact of the Covid-19 pandemic in this field of research. Regarding the respective target populations our results show that most studies (25 of 35) investigated samples with health conditions. Furthermore, only a small fraction of studies investigated children and adolescents (4 of 35) while the majority (17 of 35) investigated adults 60 years and older. Put together, these results reveal the increasing demand for remotely delivered physical fitness assessments especially in the field of telerehabilitation for older adults.

Among the physical fitness components, balance was the most frequently assessed, followed by muscular strength and muscular endurance. This focus on balance, muscular strength, and endurance aligns with the emphasis of most studies on older adults and individuals with health conditions, for whom these fitness components are particularly critical for maintaining mobility and independence [54]. Regarding validity, nearly all components showed predominantly good measures, with only a few sporadic poor measures, indicating that these components can generally be measured validly in a remote setting. However, the lack of validity measures for flexibility and speed suggests that these components may be more challenging to assess accurately in remote settings. This gap highlights the need for further research to develop and validate tests for these less commonly assessed components. In terms of reliability, the measures across all physical fitness components were predominantly good, which is encouraging for the use of these tests in both clinical and remote settings. The high amount of reliability measures of tests for balance, muscular strength, and endurance indicates that these assessments can be consistently reproduced, an essential factor for their use in ongoing health monitoring. Feasibility, as a crucial practical consideration, was rated positively across all physical fitness components. The 100% completion rates reported for the examined tests demonstrate their practicality and user-friendliness, even in remote settings. This high feasibility is particularly important as the healthcare industry increasingly embraces telehealth and remote monitoring solutions [55].

### Study qualities

The quality assessment of the studies revealed a mixed picture, suggesting potential biases across the studies. Of the non-RCTs only 10 studies received a strong global quality rating, while a substantial portion (17 studies) were rated as moderate, and five studies were rated as weak. The RCTs on the other hand showed mainly strong ratings across all the the eight items. Hereby, the study of Guidarelli et al. (2022) [31] sticks out since they used already collected data of two RCTs therefore receiving many weak and moderate ratings. For the non-RCTs moderate ratings were particularly prevalent in the components of selection bias and study design, while the blinding component was frequently rated as weak. These findings highlight methodological challenges, particularly in study design and blinding, which are critical for ensuring the internal validity of the studies. Regarding the study design it needs to be mentioned that most studies of the non-RCTs were pilot studies testing the remote assessment of physical fitness, which explains the moderate ratings. Overall, the study populations were mainly small in numbers and often recruited from hospitals or healthcare facilities, limiting their general representativeness. This aspect is discussed by many authors with the implication that the studies should be reproduced with a larger, more representative sample. Additionally, for most of the non-RCTs, it was unclear whether the assessors and participants were blinded. On the positive side, all non-RCTs received strong ratings for the data collection methods component. Additionally, almost all were rated highly for the withdrawals and drop-outs component. This point also applies to the RCTs which all reported a follow-up rate over 80%. In general the physical fitness tests used were valid and reliable leading to strong ratings. For the non-RCTs, it must be noted that the strong ratings for the confounders component should be interpreted with caution, as applying this component was challenging. As a result, ratings for this component might vary among different assessors, potentially altering the global quality ratings of the included non-RCTs.

### Implications for practitioners and future research

The findings from this analysis underscore the robustness of commonly used physical fitness tests such as the 30s-STS, TUG, and 5XSTS. These tests have been validated and shown to be reliable across different settings, making them valuable tools for assessing physical fitness, particularly in older adults and individuals with health conditions. Therefore these tests are particularly useful in tracking outcomes of home-based exercise programs for older adults, demonstrating measurable improvements in strength, balance, and mobility [56, 57]. Their adaptability makes them ideal for telehealth and remote monitoring, ensuring continuity of care during restricted mobility periods, such as the COVID-19 pandemic, as well as for individuals with mobility limitations, while supporting interventions that promote independence and reduce fall risk [56, 57]. However, the variability in the reliability of some tests, particularly those assessing balance (e.g., the Standing Balance test) or coordination, suggests that there is room for improvement in standardizing these assessments. Therefore, the current research state for the remote delivery of any physical fitness test should be thoroughly examined before use.

The underrepresentation of physical fitness tests that assess coordination, flexibility, cardiorespiratory endurance, speed, agility, and power, as well as the absence of tests for body composition and reaction time, indicates a gap in the comprehensive assessment of physical fitness. Given that these components are crucial for overall physical fitness and can therefore significantly impact the quality of life, particularly in older adults [58, 59], future research should focus on developing and validating reliable, easy-to-administer remote tests for these components.

### Limitations

To our knowledge, this is the first systematic review that highlights the evidence on the validity, reliability, and feasibility of remotely delivered physical fitness tests on a broad age spectrum. While we believe our systematic review has its strengths, it also has some limitations that need to be considered. It is possible that we omitted or excluded relevant literature during the searching and screening process. We tried to minimize this error by using a broad search strategy, involving multiple reviewers, and conducting a snowball search with the citations and reference lists of included studies. Moreover, we excluded only populations younger than one year old in our review to include as many studies as possible. However, only four studies investigated children and adolescents, limiting the significance of the results for this population. Additionally, we excluded studies that used sensors and apps for data collection, choosing to include only studies that used a videoconference format for data collection. This may have led to the exclusion of studies that remotely assessed cardiovascular endurance, body composition, agility, and reaction time. Physical fitness tests for these components are predominantly performed using devices (e.g., heart rate sensors or body composition analyzers) in conventional face-to-face settings, making it highly likely that they were assessed remotely using similar methods, leading to their exclusion from this review [60]. This is an important point that should be highlighted, as the use of fitness apps or wearable sensors (e.g. smartwatches, fitness trackers, etc.) is already widespread due to the low barriers to use and has enormous potential for the remote measurement of physical fitness, both in a clinical setting [61] and in public health. The geographical distribution of the included studies revealed an unevenly distribution across the regions of the world. Therefore, the findings of this review may not be generalizable and applicable worldwide. The majority of studies were conducted in high-income regions, specifically Europe, North America, and Australia-Oceania. A few studies were conducted in South America, Asia and one in Oceania while no study was realized in Africa. This can result in a limited understanding of how remotely delivered physical fitness tests apply to other populations with different environmental, technical, cultural, and socioeconomic conditions. Lastly, because of the heterogeneity regarding the study populations, study methods, and analysis methods in the included studies we did not perform a meta-analysis.

### Conclusion

This systematic review has highlighted the critical need for selecting appropriate physical fitness tests based on specific physical fitness components, the setting (remote or face-to-face), and the target population. The findings reveal that while tests like the 30s-STS, TUG, and 5XSTS are generally reliable and feasible, there are inconsistencies and gaps in the validity, reliability, and feasibility of many physical fitness tests, when delivered remotely. This is particularly notable in the assessment of flexibility, speed, body composition, agility, and reaction time, which are often inadequately or not tested at all.

As remote health monitoring expands, it is essential to develop and validate, reliable, and user-friendly physical fitness tests that all components of physical fitness can comprehensively be assessed. Standardization of remote delivery must be ensured for widespread adoption in both clinical and research settings. While the current findings provide a valuable foundation for clinical practice, further research and refinement are necessary to optimize these tests for more accurate and comprehensive health monitoring.

## Supplementary Information

Supplementary Material 1.

## Data Availability

The authors confirm that all data generated or analysed during this study are included in this published article or the supplementary material.

## References

[1] Ortega F, Ruiz J, Castillo M, Sjostrom M (2008) Physical fitness in childhood and adolescence: a powerful marker of health. Int J Obes. 32:1–11. 10.1038/sj.ijo.0803774 10.1038/sj.ijo.0803774 18043605

[2] Mintjens S, Menting MD, Daams JG, van Poppel MN, Roseboom TJ, Gemke RJ. Cardiorespiratory fitness in childhood and adolescence affects future cardiovascular risk factors: a systematic review of longitudinal studies. Sports Med. 2018;48:2577–605. 30144022 10.1007/s40279-018-0974-5 PMC6182463

[3] Ortega F, Cadenas-Sanchez C, Lee DC, Ruiz J, Blair S, Sui X. Fitness and Fatness as Health Markers through the Lifespan. Prog Prev Med. 2018;3:e0013. 10.1097/pp9.0000000000000013. 10.1097/pp9.0000000000000013 PMC7328664 32671316

[4] Hanssen-Doose A, Kunina-Habenicht O, Oriwol D, Niessner C, Woll A, Worth A. Predictive value of physical fitness on self-rated health: a longitudinal study. Scand J Med Sci Sports. 2021;31:56–64. 33038037 10.1111/sms.13841

[5] Lee I, Paffenbarger R, Hennekens C. Physical activity, physical fitness and longevity. Aging Clin Exp Res. 1997;9:2–11. 10.1007/BF03340123 9177581

[6] Evaristo S, Moreira C, Lopes L, Oliveira A, Abreu S, Agostinis-Sobrinho C, et al. Muscular fitness and cardiorespiratory fitness are associated with health-related quality of life: Results from labmed physical activity study. J Exerc Sci Fit. 2019;17(2):55–61. 30740134 10.1016/j.jesf.2019.01.002 PMC6353732

[7] Caspersen CJ, Powell KE, Christenson GM. Physical activity, exercise, and physical fitness: definitions and distinctions for health-related research. Public Health Rep. 1985;100(2):126. 3920711 PMC1424733

[8] Castro-Piñero J, Ortega F, Artero E, Girela-Rejón M, Mora J, Sjostrom M, et al. Assessing Muscular Strength in Youth: Usefulness of Standing Long Jump as a General Index of Muscular Fitness. J Strength Cond Res Natl Strength Cond Assoc. 2010;24:1810–7. 10.1519/JSC.0b013e3181ddb03d. 10.1519/JSC.0b013e3181ddb03d 20555277

[9] Woll A, Klos L, Burchartz A, Hanssen-Doose A, Niessner C, Oriwol D, et al. Cohort Profile Update: The Motorik-Modul (MoMo) Longitudinal Study—physical fitness and physical activity as determinants of health development in German children and adolescents. Int J Epidemiol. 2021;50(2):393–4. 10.1093/ije/dyaa281. 33709121 10.1093/ije/dyaa281

[10] Khozin S, Coravos A. Decentralized Trials in the Age of Real-World Evidence and Inclusivity in Clinical Investigations. Clin Pharmacol Ther. 2019;106. 10.1002/cpt.1441. 10.1002/cpt.1441 31013350

[11] Lai J, Widmar NO. Revisiting the digital divide in the COVID-19 era. Appl Econ Perspect Pol. 2021;43(1):458–64. 10.1002/aepp.13104 PMC7675734 33230409

[12] Heslop PA, Hurst C, Sayer AA, Witham MD. Remote collection of physical performance measures for older people: a systematic review. Age Ageing. 2023;52(1):afac327. 10.1093/ageing/afac327 PMC9889964 36721962

[13] Page MJ, McKenzie JE, Bossuyt PM, Boutron I, Hoffmann TC, Mulrow CD, et al. The PRISMA 2020 statement: an updated guideline for reporting systematic reviews. BMJ. 2021;372:n71. 10.1136/bmj.n71 PMC8005924 33782057

[14] Thomas B, Ciliska D, Dobbins M, Micucci S. A process for systematically reviewing the literature: providing the research evidence for public health nursing interventions. Worldviews Evid-Based Nurs. 2004;1(3):176–84. 17163895 10.1111/j.1524-475X.2004.04006.x

[15] Kennedy CE, Fonner VA, Armstrong KA, Denison JA, Yeh PT, O’Reilly KR, et al. The Evidence Project risk of bias tool: assessing study rigor for both randomized and non-randomized intervention studies. Syst Rev. 2019;8:1–10. 30606262 10.1186/s13643-018-0925-0 PMC6317181

[16] Botolfsen P, Helbostad JL, Moe-nilssen R, Wall JC. Reliability and concurrent validity of the Expanded Timed Up-and-Go test in older people with impaired mobility. Physiother Res Int. 2008;13(2):94–106. 18288773 10.1002/pri.394

[17] Cox NS, Alison JA, Button BM, Wilson JW, Holland AE. Assessing exercise capacity using telehealth: a feasibility study in adults with cystic fibrosis. Respir Care. 2013;58(2):286–90. 22711058 10.4187/respcare.01922

[18] Palacín-Marín F, Esteban-Moreno B, Olea N, Herrera-Viedma E, Arroyo-Morales M. Agreement between telerehabilitation and face-to-face clinical outcome assessments for low back pain in primary care. Spine. 2013;38(11):947–52. 23238489 10.1097/BRS.0b013e318281a36c

[19] Russell TG, Hoffmann TC, Nelson M, Thompson L, Vincent A. Internet-based physical assessment of people with Parkinson disease is accurate and reliable: a pilot study. J Rehabil Res Dev. 2013;50(5):643–51. 24013912 10.1682/jrrd.2012.08.0148

[20] Hwang R, Mandrusiak A, Morris NR, Peters R, Korczyk D, Russell T. Assessing functional exercise capacity using telehealth: is it valid and reliable in patients with chronic heart failure? J Telemed Telecare. 2017;23(2):225–32. 26915366 10.1177/1357633X16634258

[21] Hoenig HM, Amis K, Edmonds C, Morgan MS, Landerman L, Caves K. Testing fine motor coordination via telehealth: Effects of video characteristics on reliability and validity. J Telemed Telecare. 2018;24(5):365–72. 28350283 10.1177/1357633X17700032 PMC7523526

[22] Nicola K, Waugh J, Charles E, Russell T. The feasibility and concurrent validity of performing the Movement Assessment Battery for Children-2nd Edition via telerehabilitation technology. Res Dev Disabil. 2018;77:40–8. 29656273 10.1016/j.ridd.2018.04.001

[23] Cabrera-Martos I, Ortiz-Rubio A, Torres-Sánchez I, López-López L, Rodríguez-Torres J, Carmen Valenza M. Agreement between face-to-face and tele-assessment of upper limb functioning in patients with Parkinson disease. PM &R. 2019;11(6):590–6. 30840363 10.1002/pmrj.12001

[24] Venkataraman K, Amis K, Landerman LR, Caves K, Koh GC, Hoenig H. Teleassessment of gait and gait aids: validity and interrater reliability. Phys Ther. 2020;100(4):708–17. 31984420 10.1093/ptj/pzaa005 PMC7439231

[25] Gavazzi F, Adang L, Waldman A, Jan AK, Liu G, Lorch SA, et al. Reliability of the telemedicine application of the Gross Motor Function Measure-88 in patients with leukodystrophy. Pediatr Neurol. 2021;125:34–9. 34624609 10.1016/j.pediatrneurol.2021.09.012 PMC8629609

[26] Ogawa EF, Harris R, Dufour AB, Morey MC, Bean J. Reliability of virtual physical performance assessments in veterans during the COVID-19 pandemic. Arch Rehab Res Clin Transl. 2021;3(3):100146. 10.1016/j.arrct.2021.100146 PMC8463460 34589696

[27] Bhagat M, Mandlekar A, Verma R, Lathia T, Tanna S, Saraf A, et al. Video Call-based Fitness Assessment shows Poor Fitness in People with Type II Diabetes: Findings from Diabefly Digital Therapeutics Program. J Assoc Physicians India. 2022;70:11–2. 35833401 10.5005/japi-11001-0049

[28] Bowman A, Denehy L, Benjemaa A, Crowe J, Bruns E, Hall T, et al. Feasibility and safety of the 30-second sit-to-stand test delivered via telehealth: an observational study. PM &R. 2023;15(1):31–40. 35138036 10.1002/pmrj.12783

[29] Espin A, García-García J, Latorre Erezuma U, Aiestaran M, Irazusta J, Rodriguez-Larrad A. Videoconference-based physical performance tests: reliability and feasibility study. Int J Environ Res Public Health. 2022;19(12):7109. 35742358 10.3390/ijerph19127109 PMC9223237

[30] Fyfe JJ, Dalla Via J, Jansons P, Scott D, Daly RM. Feasibility and acceptability of a remotely delivered, home-based, pragmatic resistance ‘exercise snacking’intervention in community-dwelling older adults: a pilot randomised controlled trial. BMC Geriatr. 2022;22(1):521. 35751032 10.1186/s12877-022-03207-z PMC9233333

[31] Guidarelli C, Lipps C, Stoyles S, Dieckmann NF, Winters-Stone KM. Remote administration of physical performance tests among persons with and without a cancer history: Establishing reliability and agreement with in-person assessment. J Geriatr Oncol. 2022;13(5):691–7. 35177378 10.1016/j.jgo.2022.02.002 PMC9232927

[32] Güngör F, Ovacık U, Ertan Harputlu Ö, Yekdaneh AA, Kurt İ, Ertürk Uzunoğlu G, et al. Tele-assessment of core performance and functional capacity: reliability, validity, and feasibility in healthy individuals. J Telemed Telecare. 2024;30(6):1017–25. 35916001 10.1177/1357633X221117335

[33] Lawford BJ, Dobson F, Bennell KL, Merolli M, Graham B, Haber T, et al. Clinician-administered performance-based tests via telehealth in people with chronic lower limb musculoskeletal disorders: Test–retest reliability and agreement with in-person assessment. J Telemed and Telecare. 2024;30(8):1300–19. 10.1177/1357633X221137387. 36451551 10.1177/1357633X221137387

[34] Pelicioni PH, Waters DL, Still A, Hale L. A pilot investigation of reliability and validity of balance and gait assessments using telehealth with healthy older adults. Exp Gerontol. 2022;162:111747. 35227785 10.1016/j.exger.2022.111747

[35] Peyrusqué E, Granet J, Pageaux B, Buckinx F, Aubertin-Leheudre M. Assessing physical performance in older adults during isolation or lockdown periods: web-based video conferencing as a solution. J Nutr Health Aging. 2022;26(1):52–6. 35067703 10.1007/s12603-021-1699-y PMC8590923

[36] Aktan R, Yılmaz H, Demir İ, Özalevli S. Agreement between tele-assessment and face-to-face assessment of 30-s sit-to-stand test in patients with type 2 diabetes mellitus. Ir J Med Sci (1971-). 2023;192(5):2173–8. 10.1007/s11845-022-03238-w PMC9715279 36456718

[37] Button AM, Webster EK, Kracht CL, Hendrick C, Okely A, Chong KH, et al. Validation of remote assessment of preschool children’s anthropometrics and motor skills. Front Digit Health. 2023;5:1168618. 37519895 10.3389/fdgth.2023.1168618 PMC10373874

[38] Hoge C, Bowling CB, Dunlop-Thomas C, Pearce BD, Drenkard C, Lim SS, et al. Remote administration of physical and cognitive performance assessments in a predominantly black cohort of persons with systemic lupus erythematosus. ACR Open Rheumatol. 2023;5(9):499–507. 37582606 10.1002/acr2.11588 PMC10502850

[39] Mavronasou A, Asimakos A, Vasilopoulos A, Katsaounou P, Kortianou EA. Remote administration of the short physical performance battery, the 1-minute sit to stand, and the Chester step test in post-COVID-19 patients after hospitalization: establishing inter-reliability and agreement with the face-to-face assessment. Disabil Rehabil. 2024;44(22):5334–44. 10.1080/09638288.2023.2297928. 10.1080/09638288.2023.2297928 38156771

[40] Mehta SP, Collier PA, West KM, Workmane MC. Reproducibility and Acceptability of Short Physical Function Tests Scores Obtained via Virtual versus Face-to-Face Assessments. Crit Rev™ Phys Rehabil Med. 2023;35(1):29–44.

[41] Ng DP, Thiviyan P, Shrida S, Ng LWC. Feasibility of Conducting Sit-to-Stand Tests Using Video Consultation. Int J Telemed Appl. 2023;2023(1):8551680. 39280702 10.1155/2023/8551680 PMC11401680

[42] Núñez-Cortés R, Flor-Rufino C, Martínez-Arnau FM, Arnal-Gómez A, Espinoza-Bravo C, Hernández-Guillén D, et al. Feasibility of the 30 s Sit-to-stand test in the telehealth setting and its relationship to persistent symptoms in non-hospitalized patients with long COVID. Diagnostics. 2022;13(1):24. 36611316 10.3390/diagnostics13010024 PMC9818883

[43] Pepera G, Karanasiou E, Blioumpa C, Antoniou V, Kalatzis K, Lanaras L, et al. Tele-assessment of functional capacity through the six-minute walk test in patients with diabetes mellitus type 2: validity and reliability of repeated measurements. Sensors. 2023;23(3):1354. 36772396 10.3390/s23031354 PMC9920804

[44] Silva JDdA, Maranhão DCM, Beltrão NB, Farah BQ, Damasceno VdO, Cavalcante BR, et al. Videoconference assessment of functional and cognitive measures in Brazilian older adults: a reliability and feasibility study. Geriatr Gerontol Aging. 2023;17:1–9.

[45] Sinvani RT, Gilboa Y. Video-conference-based graphomotor examination for children: a validation study. Occup Ther J Res. 2023;43(3):351–9. 10.1177/15394492221145693 36631753

[46] Steffens D, Pocovi NC, Bartyn J, Delbaere K, Hancock MJ, Koh C, et al. Feasibility, reliability, and safety of remote five times sit to stand test in patients with gastrointestinal cancer. Cancers. 2023;15(9):2434. 37173899 10.3390/cancers15092434 PMC10177509

[47] Buckinx F, Rezoulat M, Lefranc C, Reginster JY, Bruyere O. Comparing remote and face-to-face assessments of physical performance in older adults: a reliability study. Geriatr Nurs. 2024;55:71–8. 37976558 10.1016/j.gerinurse.2023.11.004

[48] Gell NM, Dittus K, Caefer J, Martin A, Bae M, Patel KV. Remotely delivered exercise to older rural cancer survivors: a randomized controlled pilot trial. J Cancer Survivorship. 2024;18(2):596–605. 10.1007/s11764-022-01292-y PMC9662104 36374436

[49] Tütüneken YE, Buran Çirak Y, Kardeş K, Işikci B, Binbuğa R, Çetrefli E, et al. The Reliability and Validity of the Timed Up & Go Test and the 30-S Sit-To-Stand Test Performed via Tele-Assessment in Ambulatory Patients with Stroke. Meas Phys Educ Exerc Sci. 2024;28(3):236–43.

[50] Haddaway NR, Feierman A, Grainger MJ, Gray CT, Tanriver-Ayder E, Dhaubanjar S, et al. EviAtlas: a tool for visualising evidence synthesis databases. Environ Evid. 2019;8:1–10.

[51] Holmes CJ, Racette SB. The utility of body composition assessment in nutrition and clinical practice: an overview of current methodology. Nutrients. 2021;13(8):2493. 34444653 10.3390/nu13082493 PMC8399582

[52] Graveson J, Bauermeister S, McKeown D, Bunce D. Intraindividual reaction time variability, falls, and gait in old age: a systematic review. J Gerontol B Psychol Sci Soc Sci. 2016;71(5):857–64. 25969471 10.1093/geronb/gbv027

[53] Taylor ME, Lord SR, Delbaere K, Kurrle SE, Mikolaizak AS, Close JC. Reaction time and postural sway modify the effect of executive function on risk of falls in older people with mild to moderate cognitive impairment. Am J Geriatr Psychiatr. 2017;25(4):397–406. 10.1016/j.jagp.2016.10.010 28063853

[54] Cadore EL, Pinto RS, Bottaro M, Izquierdo M. Strength and endurance training prescription in healthy and frail elderly. Aging Dis. 2014;5(3):183. 24900941 10.14336/AD.2014.0500183 PMC4037310

[55] Wong MYZ, Gunasekeran DV, Nusinovici S, Sabanayagam C, Yeo KK, Cheng CY, et al. Telehealth demand trends during the COVID-19 pandemic in the top 50 most affected countries: Infodemiological evaluation. JMIR Public Health Surveill. 2021;7(2):e24445. 33605883 10.2196/24445 PMC7899203

[56] Chaabene H, Prieske O, Herz M, Moran J, Höhne J, Kliegl R, et al. Home-based exercise programmes improve physical fitness of healthy older adults: A PRISMA-compliant systematic review and meta-analysis with relevance for COVID-19. Ageing Res Rev. 2021;67:101265. 33571702 10.1016/j.arr.2021.101265

[57] Solis-Navarro L, Gismero A, Fernández-Jané C, Torres-Castro R, Solá-Madurell M, Bergé C, et al. Effectiveness of home-based exercise delivered by digital health in older adults: a systematic review and meta-analysis. Age Ageing. 2022;51(11):afac243. 10.1093/ageing/afac243 PMC9642810 36346736

[58] Dunsky A. The effect of balance and coordination exercises on quality of life in older adults: a mini-review. Front Aging Neurosci. 2019;11:318. 31803048 10.3389/fnagi.2019.00318 PMC6873344

[59] Prasad L, Fredrick J, Aruna R. The relationship between physical performance and quality of life and the level of physical activity among the elderly. J Educ Health Promot. 2021;10. 10.4103/jehp.jehp_421_20 PMC8057187 34084815

[60] Hülsdünker T, Friebe D, Giesche F, Vogt L, Pfab F, Haser C, et al. Validity of the SKILLCOURT® technology for agility and cognitive performance assessment in healthy active adults. J Exerc Sci Fit. 2023;21(3):260–7. 10.1016/j.jesf.2023.04.003 PMC10366450 37497363

[61] Muntaner-Mas A, Martinez-Nicolas A, Lavie CJ, Blair SN, Ross R, Arena R, et al. A systematic review of fitness apps and their potential clinical and sports utility for objective and remote assessment of cardiorespiratory fitness. Sports Med. 2019;49:587–600. 30825094 10.1007/s40279-019-01084-y PMC6422959

