# Supplementary material for: Remote assessment of physical fitness via videoconferencing: a systematic review
Source: BMC Sports Sci Med Rehabil. 2025 Jan 23;17:11. doi: 10.1186/s13102-024-01050-w (PMC11756083; doi:10.1186/s13102-024-01050-w)
Supplement: Supplementary file 1 — Supplementary Material 1. [file 13102_2024_1050_MOESM1_ESM.docx]

# Supplementary material: Remote assessment of physical fitness via videoconferencing: a systematic review

| **Topic** | **No.** | **Item** | **Location where item is reported** |
| --- | --- | --- | --- |
| **TITLE** |  |  |  |
| **Title** | 1 | Identify the report as a systematic review. | 1 |
| **ABSTRACT** |  |  |  |
| **Abstract** | 2 | See the PRISMA 2020 for Abstracts checklist | 1 |
| **INTRODUCTION** |  |  |  |
| **Rationale** | 3 | Describe the rationale for the review in the context of existing knowledge. | 1-2 |
| **Objectives** | 4 | Provide an explicit statement of the objective(s) or question(s) the review addresses. | 1-2 |
| **METHODS** |  |  |  |
| **Eligibility criteria** | 5 | Specify the inclusion and exclusion criteria for the review and how studies were grouped for the syntheses. | 2-3 |
| **Information sources** | 6 | Specify all databases, registers, websites, organisations, reference lists and other sources searched or consulted to identify studies. Specify the date when each source was last searched or consulted. | 2 |
| **Search strategy** | 7 | Present the full search strategies for all databases, registers and websites, including any filters and limits used. | 2 |
| **Selection process** | 8 | Specify the methods used to decide whether a study met the inclusion criteria of the review, including how many reviewers screened each record and each report retrieved, whether they worked independently, and if applicable, details of automation tools used in the process. | 2 |
| **Data collection process** | 9 | Specify the methods used to collect data from reports, including how many reviewers collected data from each report, whether they worked independently, any processes for obtaining or confirming data from study investigators, and if applicable, details of automation tools used in the process. | 2-3 |
| **Data items** | 10a | List and define all outcomes for which data were sought. Specify whether all results that were compatible with each outcome domain in each study were sought (e.g. for all measures, time points, analyses), and if not, the methods used to decide which results to collect. | 2 |
|  | 10b | List and define all other variables for which data were sought (e.g. participant and intervention characteristics, funding sources). Describe any assumptions made about any missing or unclear information. | 2 |
| **Study risk of bias assessment** | 11 | Specify the methods used to assess risk of bias in the included studies, including details of the tool(s) used, how many reviewers assessed each study and whether they worked independently, and if applicable, details of automation tools used in the process. | 3 |
| **Effect measures** | 12 | Specify for each outcome the effect measure(s) (e.g. risk ratio, mean difference) used in the synthesis or presentation of results. | 2-3 |
| **Synthesis methods** | 13a | Describe the processes used to decide which studies were eligible for each synthesis (e.g. tabulating the study intervention characteristics and comparing against the planned groups for each synthesis (item 5)). | 2-3 |
|  | 13b | Describe any methods required to prepare the data for presentation or synthesis, such as handling of missing summary statistics, or data conversions. | NA |
|  | 13c | Describe any methods used to tabulate or visually display results of individual studies and syntheses. | 2-3 |
|  | 13d | Describe any methods used to synthesize results and provide a rationale for the choice(s). If meta-analysis was performed, describe the model(s), method(s) to identify the presence and extent of statistical heterogeneity, and software package(s) used. | 2 |
|  | 13e | Describe any methods used to explore possible causes of heterogeneity among study results (e.g. subgroup analysis, meta-regression). | NA |
|  | 13f | Describe any sensitivity analyses conducted to assess robustness of the synthesized results. | NA |
| **Reporting bias assessment** | 14 | Describe any methods used to assess risk of bias due to missing results in a synthesis (arising from reporting biases). | NA |
| **Certainty assessment** | 15 | Describe any methods used to assess certainty (or confidence) in the body of evidence for an outcome. | NA |
| **RESULTS** |  |  |  |
| **Study selection** | 16a | Describe the results of the search and selection process, from the number of records identified in the search to the number of studies included in the review, ideally using a flow diagram. | 3 |
|  | 16b | Cite studies that might appear to meet the inclusion criteria, but which were excluded, and explain why they were excluded. | Supplement (4) |
| **Study characteristics** | 17 | Cite each included study and present its characteristics. | 13-16 |
| **Risk of bias in studies** | 18 | Present assessments of risk of bias for each included study. | 17 |
| **Results of individual studies** | 19 | For all outcomes, present, for each study: (a) summary statistics for each group (where appropriate) and (b) an effect estimate and its precision (e.g. confidence/credible interval), ideally using structured tables or plots. | 13-16 |
| **Results of syntheses** | 20a | For each synthesis, briefly summarise the characteristics and risk of bias among contributing studies. | NA |
|  | 20b | Present results of all statistical syntheses conducted. If meta-analysis was done, present for each the summary estimate and its precision (e.g. confidence/credible interval) and measures of statistical heterogeneity. If comparing groups, describe the direction of the effect. | NA |
|  | 20c | Present results of all investigations of possible causes of heterogeneity among study results. | NA |
|  | 20d | Present results of all sensitivity analyses conducted to assess the robustness of the synthesized results. | NA |
| **Reporting biases** | 21 | Present assessments of risk of bias due to missing results (arising from reporting biases) for each synthesis assessed. | NA |
| **Certainty of evidence** | 22 | Present assessments of certainty (or confidence) in the body of evidence for each outcome assessed. | NA |
| **DISCUSSION** |  |  |  |
| **Discussion** | 23a | Provide a general interpretation of the results in the context of other evidence. | 6 |
|  | 23b | Discuss any limitations of the evidence included in the review. | 7 |
|  | 23c | Discuss any limitations of the review processes used. | 7 |
|  | 23d | Discuss implications of the results for practice, policy, and future research. | 7 |
| **OTHER INFORMATION** |  |  |  |
| **Registration and protocol** | 24a | Provide registration information for the review, including register name and registration number, or state that the review was not registered. | 2 |
|  | 24b | Indicate where the review protocol can be accessed, or state that a protocol was not prepared. | NA |
|  | 24c | Describe and explain any amendments to information provided at registration or in the protocol. | NA |
| **Support** | 25 | Describe sources of financial or non-financial support for the review, and the role of the funders or sponsors in the review. | 8 |
| **Competing interests** | 26 | Declare any competing interests of review authors. | 8 |
| **Availability of data, code and other materials** | 27 | Report which of the following are publicly available and where they can be found: template data collection forms; data extracted from included studies; data used for all analyses; analytic code; any other materials used in the review. | 8 |

**Supplementary Table 1.** PRISMA 2020 Checklist.[^1^](#_bookmark0)

Author & Year Reason for exclusion

Abbas et al., 2021[^2^](#_bookmark1) no physical fitness tests

Anghelescu, 2022[^3^](#_bookmark2) not videoconference based

Baque et al., 2017[^4^](#_bookmark3) not videoconference based

Chen et al., 2021[^5^](#_bookmark4) test methodology not reviewed

Chow et al., 2020[^6^](#_bookmark5) not videoconference based Cristinziano et al., 2022[^7^](#_bookmark6) not videoconference based Dobkin, 2017[^8^](#_bookmark7) not videoconference based Espin et al., 2023[^9^](#_bookmark8) test methodology not reviewed Finkelstein et al., 2020[^10^](#_bookmark9) no physical fitness tests

Hale & Kimberley, 2014[^11^](#_bookmark10) not videoconference based Isernia et al., 2020[^12^](#_bookmark11) test methodology not reviewed Kanai et al., 2016[^13^](#_bookmark12) not videoconference based Kelly & Moran, 2010[^14^](#_bookmark13) not videoconference based Lipsmeier et al., 2022[^15^](#_bookmark14) not videoconference based Ozturk & Duruturk, 2022[^16^](#_bookmark15) no physical fitness tests

Patel et al., 2021[^17^](#_bookmark16) test methodology not reviewed Prieto et al., 2022[^18^](#_bookmark17) not videoconference based Rees-Punia et al., 2021[^19^](#_bookmark18) not videoconference based Robinson et al., 2015[^20^](#_bookmark19) no physical fitness tests

Saitoh et al., 2022[^21^](#_bookmark20) not videoconference based Spinosa et al., 2020[^22^](#_bookmark21) not videoconference based Young et al., 2021[^23^](#_bookmark23) not videoconference based

**Supplementary Table 2.** List of studies excluded at full-text review stage.

| Physical fitness component (Total number of tests) | | | | | | | | | | | |
| --- | --- | --- | --- | --- | --- | --- | --- | --- | --- | --- | --- |
| Physical fitness test  (Total number of studies) | Cardiorespiratory  endurance (4) | Muscular endurance (12) | Muscular strength (16) | Body composition (0) | Flexibility (3) | Agility (1) | Balance (17) | Coordination (8) | Speed (3) | Power (3) | Reaction time (0) |
| 30s-STS (14) |  | X | X |  |  |  | X |  |  | X |  |
| 5XSTS (11) |  |  | X |  |  |  | X |  |  |  |  |
| TUG (11) |  |  | X |  |  |  | X |  |  |  |  |
| 4m-WT (6) |  |  |  |  |  |  |  |  | X |  |  |
| SB (4) |  |  |  |  |  |  | X |  |  |  |  |
| 6M-WT (3) | X |  |  |  |  |  |  |  |  |  |  |
| UB (3) |  |  |  |  |  |  | X |  |  |  |  |
| 10XSTS (2) |  |  | X |  |  |  |  |  |  | X |  |
| 1M-STS (2) |  | X | X |  |  |  |  |  |  |  |  |
| 2M-ST (2) | X |  |  |  |  |  |  |  |  |  |  |
| BBS (2) |  |  |  |  |  |  | X |  |  |  |  |
| FR (2) |  |  |  |  |  |  | X |  |  |  |  |
| FT (2) |  |  |  |  |  |  |  | X |  |  |  |
| 1M-PU (1) |  | X | X |  |  |  |  |  |  |  |  |
| 1M-SU (1) |  | X | X |  |  |  |  |  |  |  |  |
| 30s-AC (1) |  | X | X |  |  |  |  |  |  |  |  |
| 360-TT (1) |  |  |  |  |  |  | X |  |  |  |  |
| 3M-ST (1) | X |  |  |  |  |  |  |  |  |  |  |
| 5m-FW (1) |  |  |  |  |  |  |  |  | X |  |  |
| 9-PB (1) |  |  |  |  |  |  |  | X |  |  |  |
| CR (1) |  |  |  |  |  |  |  | X |  |  |  |
| CRT (1) |  | X | X |  |  |  |  |  |  |  |  |
| CST (1) | X |  |  |  |  |  |  |  |  |  |  |
| CU (1) |  | X | X |  |  |  |  |  |  |  |  |
| DGI (1) |  |  |  |  |  |  | X |  |  |  |  |
| ETUG (1) |  |  | X |  |  |  | X |  |  |  |  |
| FGA (1) |  |  |  |  |  |  | X |  |  |  |  |
| FN (1) |  |  |  |  |  |  |  | X |  |  |  |
| GIFT (1) |  |  |  |  |  |  |  | X |  |  |  |
| GMFM-88 (1) |  |  |  |  |  |  |  | X |  |  |  |
| KPU (1) |  | X | X |  |  |  |  |  |  |  |  |
| LB (1) |  |  | X |  |  |  |  |  |  |  |  |
| LR (1) |  |  |  |  |  |  | X |  |  |  |  |
| MABC2 (1) |  |  |  |  |  |  | X | X |  |  |  |
| MPU (1) |  | X | X |  |  |  |  |  |  |  |  |
| POMA-G (1) |  |  |  |  |  |  | X |  |  |  |  |
| PT (1) |  | X | X |  |  |  |  |  |  |  |  |
| SAR (1) |  |  |  |  | X |  |  |  |  |  |  |
| SCT (1) |  |  |  |  |  |  |  |  | X |  |  |
| SITFE (1) |  | X |  |  |  |  |  |  |  |  |  |
| SLJ (1) |  |  |  |  |  |  |  |  |  | X |  |
| SLS (1) |  |  |  |  |  |  | X |  |  |  |  |
| SoT (1) |  | X |  |  |  |  |  |  |  |  |  |
| SRT (1) |  |  |  |  | X |  | X |  |  |  |  |
| ST (1) |  |  |  |  |  |  | X |  |  |  |  |
| S-TUG (1) |  |  |  |  |  | X |  | X |  |  |  |
| V-SR (1) |  |  |  |  | X |  |  |  |  |  |  |
| WS (1) |  |  | X |  |  |  |  |  |  |  |  |

**Supplementary Table 3.** 30s-STS = 30 second sit-to-stand test; 5XSTS = 5-times sit-to-stand test; TUG = Timed up and go; 4m-WT = 4-meter walk test; SB = Standing balance; 6M-WT = 6-minute walk test; UB = Unipedal balance test; 10XSTS = 10-times sit-to-stand test; 1M-STS = 1-minute sit-to-stand test; 2M-ST = 2-minute step test; BBS = Berg balance scale; FR = Functional reach test; FT = Finger-tapping test; 1M-PU = 1-minute push-up test; 1M-SU = 1-minute sit-up test; 30s-AC =

30-second arm curl test; 360-TT = 360° Turn test; 3M-ST = 3-minute step test; 5m-FW = 5-meter fast-paced walk; 9-PB = 9-hole pegboard test; CR = Coin rotation task; CRT = Calf raise test; CST = Chester step test; CU = Curl-up test; DGI =

Dynamic gait index; ETUG = Expanded timed up and go; FGA = Functional gait assessment; FN = Finger-nose test; GIFT = Gilboa functional test; GMFM-88 = Gross motor function measure-88; KPU = Kneeling push-up test; LB = Lateral bridge test; LR = Lateral reach test; MABC2 = Movement Assessment Battery for Children – Second Edition; MPU = Modified push-up test; POMA-G = Tinetti Performance-Oriented Mobility Assessment Gait Scale; PT = Plank test; SAR = Stand and reach; SCT

= Stair climb test; SITFE = Shirado–Ito trunk flexor endurance test; SLJ = Standing long jump; SLS = Single leg stance; SoT = Sorensen test; SRT = Sitting and rising test; ST = Step test; S-TUG = Supine-timed up and go; V-SR = V-sit and reach test; WS

= Wall sit test.

# References

1. Page, M. J. *et al.* The prisma 2020 statement: an updated guideline for reporting systematic reviews. *bmj* **372** (2021).
2. Abbas, A. *et al.* Computer vision-based assessment of motor functioning in schizophrenia: use of smartphones for remote measurement of schizophrenia symptomatology. *Digit. Biomarkers* **5**, 29–36 (2021).
3. Anghelescu, A. Telerehabilitation: A practical remote alternative for coaching and monitoring physical kinetic therapy in patients with mild and moderate disabling parkinson’s disease during the covid-19 pandemic. *Park. Dis.* **2022**, 4370712 (2022).
4. Baque, E., Barber, L., Sakzewski, L. & Boyd, R. N. Randomized controlled trial of web-based multimodal therapy for children with acquired brain injury to improve gross motor capacity and performance. *Clin. rehabilitation* **31**, 722–732 (2017).
5. Chen, S.-C., Lin, C.-H., Su, S.-W., Chang, Y.-T. & Lai, C.-H. Feasibility and effect of interactive telerehabilitation on balance in individuals with chronic stroke: a pilot study. *J. neuroengineering rehabilitation* **18**, 1–11 (2021).
6. Chow, D. H., Cheng, W. H. & Tam, S. S. A video-based classification system for assessing locomotor skills in children. *J.* *sports science & medicine* **19**, 585 (2020).
7. Cristinziano, M. *et al.* Telerehabilitation during covid-19 lockdown and gross motor function in cerebral palsy: an observational study. *Eur. journal physical rehabilitation medicine* **58**, 592 (2022).
8. Dobkin, B. H. A rehabilitation-internet-of-things in the home to augment motor skills and exercise training. *Neurorehabili-* *tation neural repair* **31**, 217–227 (2017).
9. Espin, A. *et al.* Effects of a videoconference-based therapeutic exercise intervention on the musculoskeletal pain of eldercare workers: protocol for the revieew randomized controlled trial. *BMC Musculoskelet. Disord.* **24**, 463 (2023).
10. Finkelstein, J. *et al.* Usability of remote assessment of exercise capacity for pulmonary telerehabilitation program. In

*Integrated Citizen Centered Digital Health and Social Care*, 72–76 (IOS Press, 2020).

1. Hale, L. & Donovan, K. A video-based balance measure for people with intellectual disability. *J. Intellect. Dev. Disabil.*

**39**, 206–213 (2014).

1. Isernia, S. *et al.* Effects of an innovative telerehabilitation intervention for people with parkinson’s disease on quality of life, motor, and non-motor abilities. *Front. neurology* **11**, 846 (2020).
2. Kanai, A. *et al.* Use of the sit-to-stand task to evaluate motor function of older adults using telemetry. *BMC geriatrics* **16**, 1–10 (2016).
3. Kelly, L. E. & Moran, T. E. The effectiveness of a web-based motor skill assessment training program. *ICHPER-SD J. Res.*

**5**, 48–53 (2010).

1. Lipsmeier, F. *et al.* A remote digital monitoring platform to assess cognitive and motor symptoms in huntington disease: cross-sectional validation study. *J. Med. Internet Res.* **24**, e32997 (2022).
2. Ozturk, B. & Duruturk, N. Effect of telerehabilitation applied during covid-19 isolation period on physical fitness and quality of life in overweight and obese individuals. *Int. J. Obes.* **46**, 95–99 (2022).
3. Patel, J. *et al.* Effects of supervised exercise-based telerehabilitation on walk test performance and quality of life in patients in india with chronic disease: Combatting covid-19. *Int. journal telerehabilitation* **13** (2021).
4. Prieto, L. A., Meera, B., Katz, H. & Columna, L. A feasibility trial for virtual administration of the test of gross motor development-3 for children with autism spectrum disorders during the covid-19 pandemic. *Adapt. Phys. Activity Q.* **39**, 446–455 (2022).
5. Rees-Punia, E., Rittase, M. H. & Patel, A. V. A method for remotely measuring physical function in large epidemiologic cohorts: Feasibility and validity of a video-guided sit-to-stand test. *PloS one* **16**, e0260332 (2021).
6. Robinson, L. E. *et al.* Development and reliability testing of a video-based instrument designed to assess perceived motor skill competence in children. In *JOURNAL OF SPORT & EXERCISE PSYCHOLOGY*, vol. 37, S12–S12 (HUMAN KINETICS PUBL INC 1607 N MARKET ST, PO BOX 5076, CHAMPAIGN, IL 61820 . . . , 2015).
7. Saitoh, M. *et al.* Remote cardiac rehabilitation in older cardiac disease: a randomized case series feasibility study. *Cardiol.* *Res.* **13**, 57 (2022).
8. Spinosa, R. M. d. O. *et al.* Comparing live and digital augmented reality models for demonstrating two motor skills from the test of gross motor development—second edition: Tgmd-2. *Percept. Mot. Ski.* **127**, 386–400 (2020).
9. Young, A., Healy, S., Silliman-French, L. & Brian, A. A pilot study of a parent-mediated, web-based motor skill intervention for children with down syndrome: Project skip. *Adapt. Phys. Activity Q.* **38**, 452–473 (2021).
